# Inflammation-suppressing cornea-in-a-syringe with anti-viral GF19 peptide promotes regeneration in HSV-1 infected rabbit corneas

**DOI:** 10.1038/s41536-024-00355-1

**Published:** 2024-03-01

**Authors:** Egidijus Simoliunas, Inés Ruedas-Torres, Yolanda Jiménez-Gómez, Elle Edin, Mozhgan Aghajanzadeh-Kiyaseh, Mostafa Zamani-Roudbaraki, Rimvydas Asoklis, Milda Alksne, Neethi C. Thathapudi, Bijay K. Poudel, Ieva Rinkunaite, Kasparas Asoklis, Monika Iesmantaite, Laura Ortega-Llamas, Almantas Makselis, Marcelo Munoz, Daiva Baltriukiene, Virginija Bukelskiene, Jaime Gómez-Laguna, Miguel González-Andrades, May Griffith

**Affiliations:** 1 Department of Biological Models, Institute of Biochemistry, Life Sciences Center, Vilnius University, Vilnius, Lithuania; 2 Department of Anatomy and Comparative Pathology and Toxicology, Pathology and Immunology Group (UCO-PIG), UIC Zoonosis y Enfermedades Emergentes ENZOEM, University of Córdoba, International Excellence Agrifood Campus ‘CeiA3’, 14014 Córdoba, Spain; 3 Maimonides Biomedical Research Institute of Cordoba (IMIBIC), Department of Ophthalmology, Reina Sofia University Hospital and University of Cordoba, 14004 Cordoba, Spain; 4 Department of Ophthalmology and Institute of Biomedical Engineering, University of Montreal, Montrea, QC Canada; 5 Maisonneuve-Rosemont Hospital Research Centre, Montreal, QC Canada; 6 Department of Ophthalmology, Vilnius University Hospital, Vilnius, Lithuania; 7 Heart Institute, University of Ottawa, Ottawa, ON Canada

**Keywords:** Regenerative medicine, Translational research, Implants

## Abstract

Pathophysiologic inflammation, e.g., from HSV-1 viral infection, can cause tissue destruction resulting in ulceration, perforation, and ultimately blindness. We developed an injectable Cornea-in-a-Syringe (CIS) sealant-filler to treat damaged corneas. CIS comprises linear carboxylated polymers of inflammation-suppressing 2-methacryloyloxyethyl phosphorylcholine, regeneration-promoting collagen-like peptide, and adhesive collagen-citrate glue. We also incorporated GF19, a modified anti-viral host defense peptide that blocked HSV-1 activity in vitro when released from silica nanoparticles (SiNP-GF19). CIS alone suppressed inflammation when tested in a surgically perforated and HSV-1-infected rabbit corneal model, allowing tissue and nerve regeneration. However, at six months post-operation, only regenerated neocorneas previously treated with CIS with SiNP-GF19 had structural and functional features approaching those of normal healthy corneas and were HSV-1 virus-free. We showed that composite injectable biomaterials can be designed to allow regeneration by modulating inflammation and blocking viral activity in an infected tissue. Future iterations could be optimized for clinical application.

## Introduction

Inflammation is critical to the body’s ability to heal itself. However, uncontrolled, intense, acute or chronic organ-specific inflammation due to injury or disease can result in organ damage and failure^1^. This is true even for the usually avascular and immune-privileged human cornea. The cornea is the transparent front of the eye that focuses two-thirds of light entering for vision^2^. If transparency is lost due to damage or disease, cornea transplantation is the method of choice for eyesight restoration. While corneal transplantation is very successful in non-inflamed corneas due to tissue immune privilege, the privilege is lost as a consequence of severe infections or injuries in inflamed and neovascularized corneas. Severe corneal inflammation or keratitis resulting from injuries or disease is, therefore, a leading cause of blindness worldwide due to the destruction of corneal tissue, resulting in ulceration or even perforation^3^. The graft failure rate in these inflamed corneas is as high as 70% despite maximum immune suppression^4^. With each subsequent graft, the failure rate increases as these high-risk patients move closer to permanent blindness^5^. Controlling inflammation is therefore crucial to vision restoration.

Traumatic and non-traumatic, often infectious, causes of corneal perforations are amongst inflammation’s most severe outcomes. They are medical emergencies, and cyanoacrylate glue is used to seal and save eyeball integrity. However, multiple applications and follow-on corneal transplantation are generally needed to restore vision^6^. Other commercial sealants such as polyethylene glycol (PEG)-based ReSure and Ocuseal are also sub-optimal^7–9^, while fibrin glue forms a poor seal and degrades rapidly^10^. Other sealants developed include ones based on chitosan^11^, gelatin-methacrylate (GELGYM and GelCORE)^12,13^ and collagen-based peptides^14^, most requiring photocrosslinking to work.

Our aim was to bioengineer an injectable Cornea-in-a-Syringe (CIS) cell-free biomaterial that can seal and fill perforations in inflamed corneas and promote their regeneration, avoiding the need for corneal transplantation and high risk of graft failure. As patients with inflamed, ulcerated or perforated corneas are generally photophobic^15^, our CIS fills and seals the perforation, and spontaneously gels into a solid hydrogel scaffold without need for photocrosslinking. Our biomimetic materials comprise a collagen-like peptide (CLP)^16^ to promote regeneration, and a pre-polymerized artificial phospholipid based on 2-methacryloyloxyethyl phosphorylcholine (MPC)^17,18^ with inflammation-suppressing properties^18^. As monomeric MPC is toxic, we designed and synthesized a linear carboxylated phosphorylcholine polymer (LCPP). The CIS is applied from two syringes containing CLP-LCPP and a modified collagen-citrate glue^19^.

When inflammation results from infection, it is not always possible to clear the infection before repairing the damage, particularly in emergencies like corneal perforation. Therefore, we expanded our CIS’s capacity to release bioactive factors or drugs by incorporating drug-delivering nanoparticles to treat a concurrent infection. *Herpes Simplex Virus* serotype 1 (HSV-1) corneal infection initiates a chronic immune-inflammatory response, Herpes Simplex Keratitis (HSK), which is the main cause of infectious blindness in the developed world. Globally, 1.5 million cases and 40,000 new cases are reported annually^20^. HSK usually results from reactivation of HSV-1, which establishes latency in the trigeminal ganglia of the brain after the initial infection. Cornea latency has also been reported^21^. Recurrent infections, likely from viral reactivation, can result in intense inflammation destroying corneal tissue, causing ulceration or perforation. While corneal transplantation is the current treatment for damaged corneas, even with immunosuppression, surgery or any trauma to the eye can reactivate HSV-1. Peri-surgical reactivation resulting in graft failure for over 18% of corneal transplantations is therefore challenging^22^. Anti-viral drugs (e.g., Acyclovir or Ganciclovir) efficaciously clear viruses from the body by preventing their replication but are inefficient prophylactics with known adverse side effects^23^. The human body produces several cationic human defense peptides (cHDPs) that protect against infection^24^, including a cathelicidin, LL37. The body’s corneal epithelium and other epithelia produce LL37, the only human cathelicidin^25^. LL37 can target HSV-1 viruses directly by permeabilizing their viral external lipid membrane^25^. LL37 is cytotoxic, but its smaller fragments with anti-viral activity are more biocompatible. GF17, a 17 amino acid LL37 fragment containing the crucial antimicrobial region (residues 17–29) and extra amino acids, N-L-V, has demonstrated anti-viral activity against the human immunodeficiency virus (HIV)^26^. GF17 also showed direct anti-viral activity against Zika, another enveloped neurotropic virus, by disruption of the viral envelope; GF17 can also act through the interferon pathway by upregulating type I interferon^27^.

In this study, we added two cell-penetrating amino acids to GF17 to enhance its ability to disrupt viral envelopes and named this modified peptide GF19. GF19 (GFKRIVQRIKDFLRNLVKL-NH_2_) loaded in silica nanoparticles (SiNP-GF19) was added to the CIS. The resulting CIS sealant-filler with anti-inflammation properties together with the additional anti-viral properties was tested in surgically perforated rabbit corneas that were infected peri-surgically with the McKrae strain of HSV-1 isolated from an HSK patient^28^ to simulate corneal perforations arising from inflammation due to infection.

## Results

### CIS characterization

**Fig. 1:**
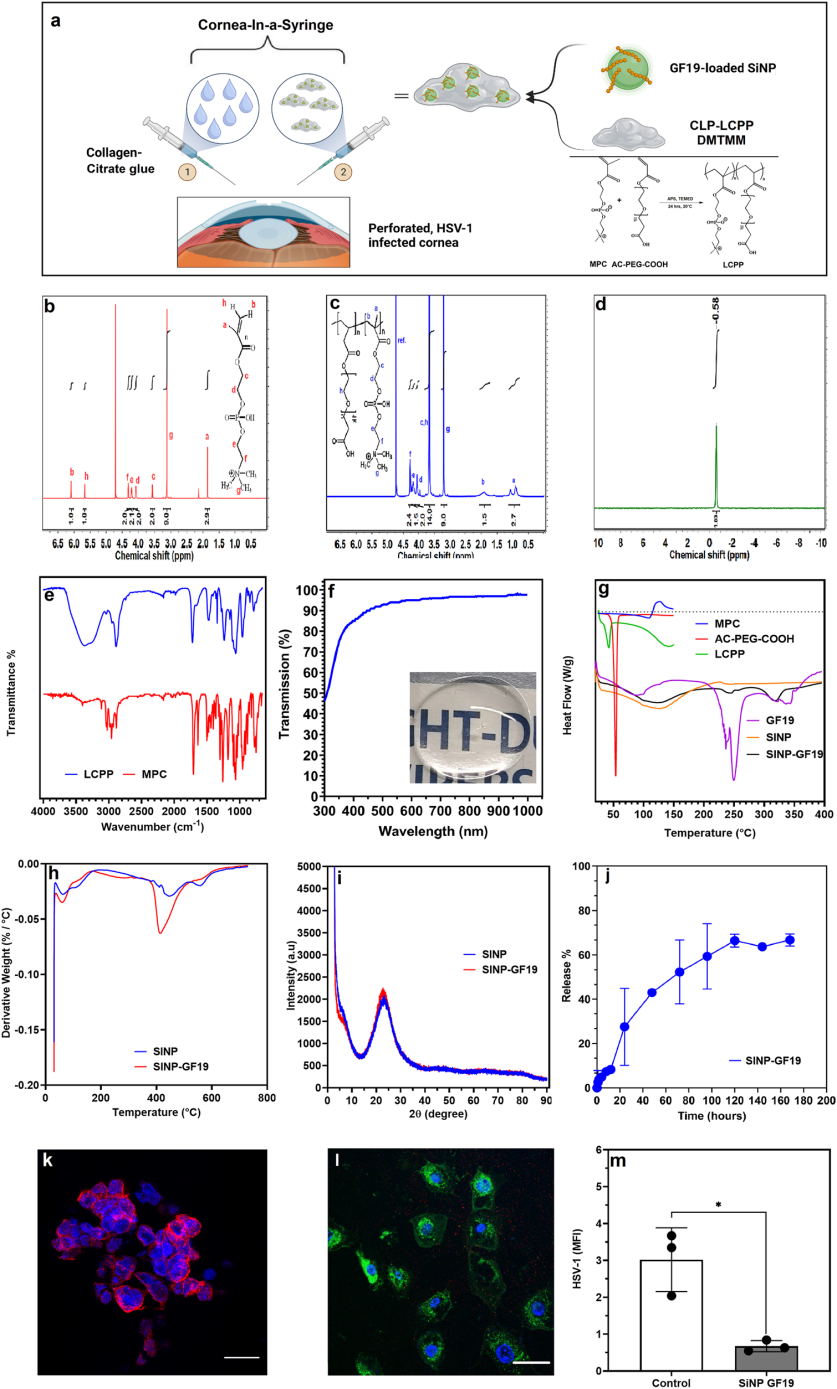
Hydrogel and SiNP-GF19 characterization. **a** Preparation of SiNP-GF19 loaded CIS and LCPP. **b** ^1^H NMR spectrum of MPC. **c** ^1^H NMR spectrum of LCPP. **d** ^31^P NMR spectrum of LCPP. **e** FTIR spectra of MPC and LCPP. **f** Transparency of CLP-LCPP/citrate glue hydrogel shown by transmission of light through the hydrogel from the UV range through visible light (380 to 700 nm). *n* = 4 samples; average was reported. An image of a transparent CIS hydrogel is shown. **g** DSC curves of AC-PEG-COOH, MPC, LCPP, GF19, SiNP, and SiNP-GF19. **h** Derivative TGA curves of SiNP and SiNP-GF19. **i** XRD diffraction patterns of SiNP and SiNP-GF19. **j** GF19 release profile from SiNPs; *n* = 3 samples, mean ± SD. **k** HSV-1 infected and untreated human corneal epithelial cells (HCECs) at 48 h post-infection showing anti-HSV-1 antibody-stained virus (red) and cell nuclei were stained with DAPI (blue). Scale bar, 33 µm. **l** HCECs treated with SiNP loaded with FITC-labeled GF19 (green) for 1 h prior to HSV-1 infection. The cells were inoculated with virus for 1 h, after which both peptide and virus were removed. Cells are shown at 48 h post-infection. All cell nuclei were stained with DAPI (blue). Scale bars, 33 µm, **m** Mean fluorescence intensity (MFI) of HSV-1 staining in virus-infected untreated *vs*. SiNP-GF19-treated cells. *P = 0.098 by Student’s *t*-test, with statistical significance set at *P* ≤ 0.05. (*n* = 3 repeats per group, mean ± SD).

The preparation of CIS containing SiNP-GF19 for injection into the cornea, showing the free radical polymerization scheme used to synthesize LCPP is summarized in Fig. 1a. The ^1^H NMR spectrum of MPC in Fig. 1b showed two peaks in the 5.5–6.2 ppm range from the vinylic hydrogen of monomeric MPC. After polymerization and formation of LCPP (Fig. 1c), one of these two peaks disappeared while the other one shifted to 1.9 ppm, confirming that no unsaturated bonds were left in the LCPP. There is also a peak at 3.7 ppm that can be assigned to the methylene group of PEG, while the singlet peak at 3.2 ppm can be assigned to three methyl groups of MPC, confirming the successful polymerization of MPC and AC-PEG-COOH to give LCPP. Moreover, the ^31^P NMR spectrum of LCPP showed a single phosphorus peak at 0.58 ppm that was assigned to MPC, further confirming the synthesis (Fig. 1d).

The FTIR spectrum of MPC has two characteristic phosphorus peaks at 1060 and 1253 cm^−1^ that are also present in LCPP (Fig. 1e). However, LCPP showed an additional distinctive characteristic peak from the methylene groups of PEG around 2850 cm^−1^. The intensity ratio of this peak, in comparison to other peaks on the LCPP curve, is higher than the same one in the FTIR spectrum of MPC. Transmission of light through the CLP-LCPP hydrogel (Fig. 1f) was above 85% across the visible spectrum of 380 to 700 nm, matching the characteristics of the healthy corneas^29^. Differential Scanning Calorimetry (DSC) of AC-PEG-COOH (Fig. 1g) showed an endothermic peak of AC-PEG-COOH at around 55 °C, but no peaks up to 150 °C for MPC. However, in LCPP, the endothermic peak shifted to 45 °C, due to the incorporation of MPC into PEG during LCPP synthesis.

Bursting pressure testing^16^ of CIS to determine its mechanical strength and adhesivity to corneal tissue was performed. Excised pig corneas with 5 mm diameter circular wounds down to 50% depth with a 1 mm central full-thickness perforation filled with CIS that gelled in situ tolerated an average pressure of 47.1 ± 4.3 mmHg (Supplementary Fig. 1a). This pressure was more than double the normal intraocular pressure of the human eyeball of 16 mmHg (range: 12–22 mmHg)^30^. The water content of the CIS was 95.3 ± 0.9%, higher than that of the human cornea at 78–92%^31^. The hydrogel was biocompatible and supported growth of an immortalized human cornea epithelial cell line (Supplementary Fig. 2)^32^.

### GF19-containing silica nanoparticles and composite CIS

GF19 was loaded into solid silica nanoparticles (SiNPs) as we previously described for its parent peptide, LL37^33^, and in turn, incorporated into CIS as shown in Fig. 1a to make CIS+SiNP-GF19. GF19 was also incorporated into a commercial ointment to extend the availability of GF19 (CIS+SiNP-GF19+ointment).

Although SiO_2_ does not show any weight loss below 730 °C by derivative thermogravimetric analysis (TGA)^34^, the curves of SiNP and SiNP-GF19 containing Triton X-100 showed weight reduction (Fig. 1h). However, the reduction in weight in the SiNP-GF19 was 3% greater than for SiNP, the difference accounting for the amount of GF19 loaded into the SiNP. DSC curves of GF19, SiNP and SiNP-GF19 (Fig. 1g) showed endometric peaks at 248 and 342 °C for GF19, indicating melting and degradation points. SiNP had no peaks above 150 °C. In the SiNP-GF19 curve, both peaks of GF19 had shifted to slightly lower temperatures, confirming SiNP-GF19 interaction. X-ray diffraction patterns of SiNP and SiNP-GF19 showed a single peak around 22° that is characteristic for amorphous silicon dioxide (SiO_2;_ Fig. 1i), indicating that SiNPs remained amorphous after loading of GF19.

The encapsulation efficiency (EE) of GF19 was 96.60 ± 1.17% (Supplementary Table 6). The cumulative release of GF19 from peptide-loaded SiNP at a physiological pH (7.4) and 35 °C, the temperature of the human cornea^35^ (Fig. 1j) was gradual, reaching 60 and 67% after four and seven days, respectively. A strong electrostatic interaction (hydrogen bond) between Si–OH (silanol groups) and Si–O–Si (siloxide groups) within the SiNPs and different polar functional groups of amino acids in GF19 most likely resulted in diffusion-controlled release of GF19 rather than an initial burst release. The average particle size of SiNP and SiNP-GF19 were 131.5 ± 68.8 nm and 165.3 ± 64.5 nm, respectively (Supplementary Fig. 1b, Supplementary Table 6). The size increase was due to the presence of GF19 within the SiNPs. After incorporation into SiNP, the zeta potential of the combined SiNP-GF19 increased from −21.57 ± 0.84 to −15.05 ± 1.11 mV, due to the presence of the cationic peptides (Supplementary Table 6).

SiNP-GF19, and CIS+SiNP-GF19 supported the adhesion and proliferation of HCECs in cultures (Supplementary Fig. 2). Importantly, the released GF19 retained its activity. When HCECs were infected with 0.5 multiplicity of infection of HSV-1, the cells showed cytopathic effects – rounded and dead at 48 h post-infection (Fig. 1k). However, when treated with 5 µM FITC-GF19 released from SiNPs one hour prior to infection, the HCECs remained spread and adherent at 48 h post-infection. GF19 was localized to the cells while HSV-1 particles were excluded (Fig. 1l). However, when GF19 was given at the time of infection, the HCECs were infected (i.e., HSV-1 was seen within the cells) but they remained adherent at 48 h post-infection (Supplementary Fig. 3). These results showed that GF19 slowed down the infection but did not prevent it. HSV-1 activity, expressed in mean fluorescence intensity (MFI), was significantly decreased by the SiNP-GF19 treatment (Fig. 1m).

### In vivo rabbit HSV-1-perforation model and wound healing

**Fig. 2:**
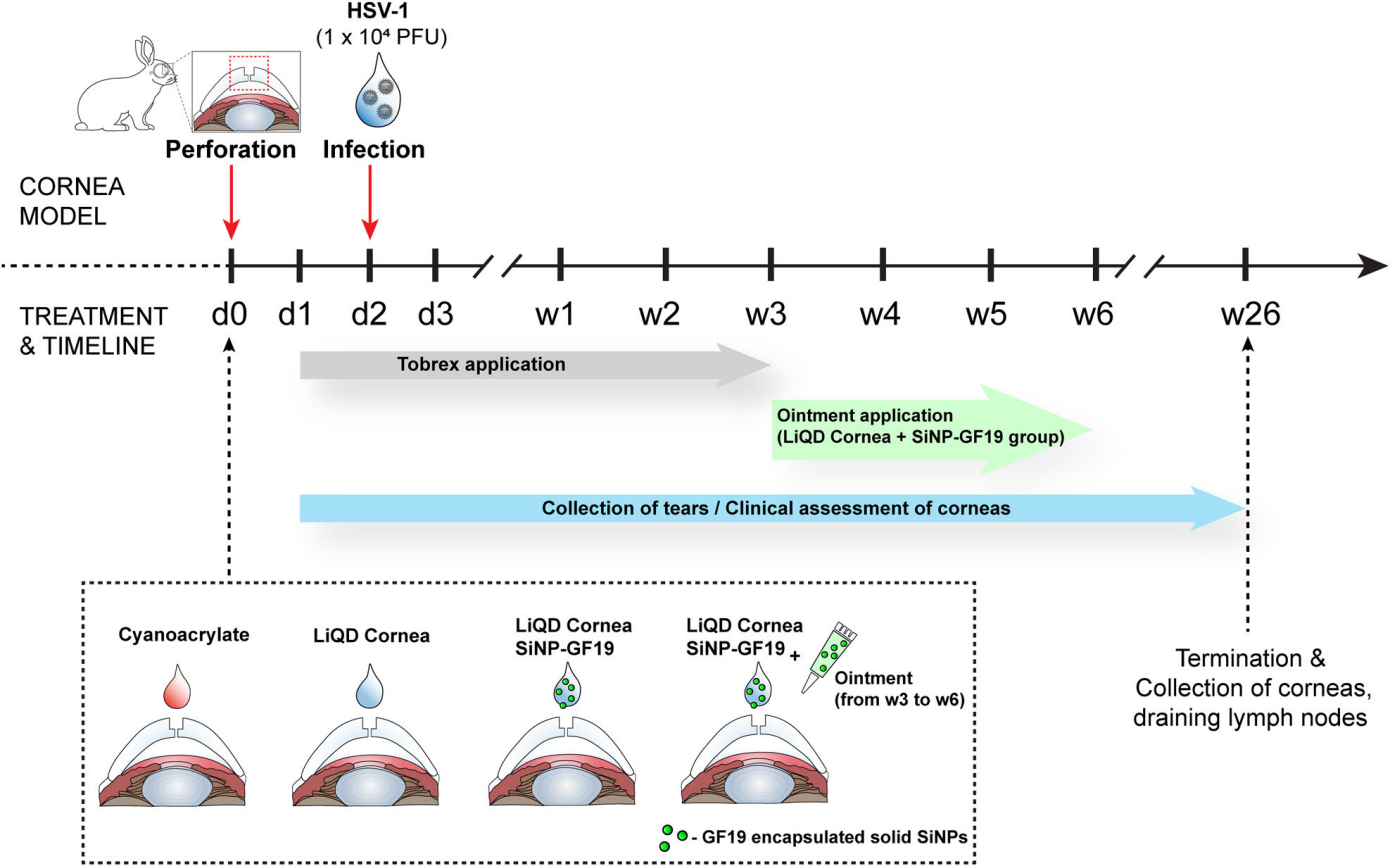
Timeline of the in vivo rabbit study. The left eye of each animal received a surgically created perforation that was sealed and filled with one of cyanoacrylate, CIS, or CIS containing SiNP-GF19 (d0). On the second day after surgery (d2), the operated eyes were inoculated with 1×10^4^ PFU HSV-1 to create a peri-surgical infection. All operated eyes were treated daily with tobramycin antibiotic (Tobrex) ointment for 3 weeks after surgery. The rabbit eyes of the CIS+SiINP-GF19+oint. group were additionally treated with an ointment containing SiNP-GF19 for an additional three weeks after completion of the antibiotic course. Tear collection and healing of the left eyes were monitored as indicated over the 26-week post-operation follow-up.

**Fig. 3:**
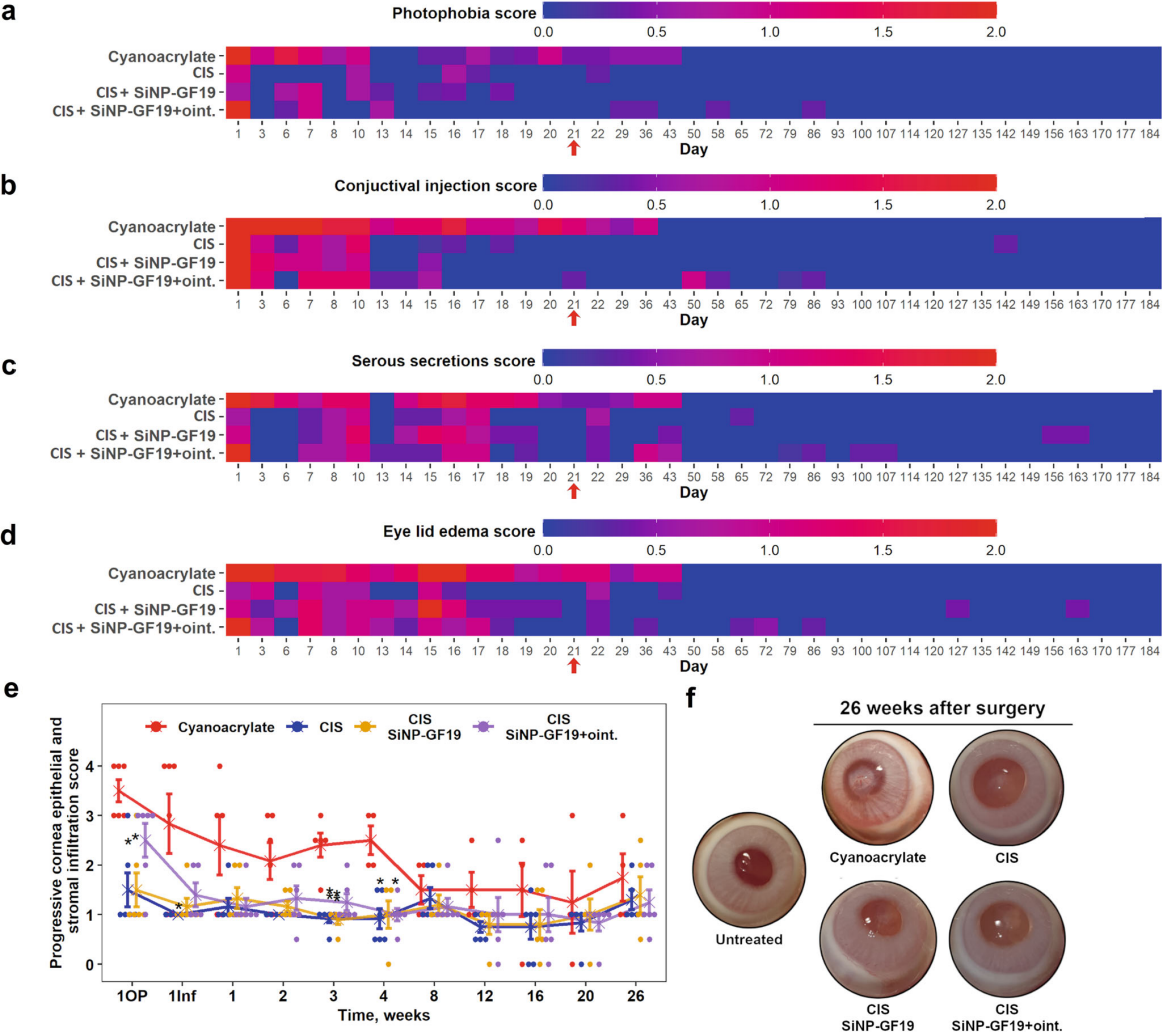
Clinical follow-up of perforated and HSV-1 infected rabbit corneas after various treatments. Heat maps show the clinical progression of corneal wound healing of the following parameters: **a** photophobia, **b** conjunctivitis, **c** tearing, and **d** periorbital edema. Blue corresponds to a healthy eye and red to a clinical worsening of the eye. The red arrows in the plots indicate when the CIS+SiNP-GF19+oint. group received additional ointment containing SiNP-GFP. **e** Infiltration of immune cells recorded at different time points: 1OP - the day after surgical perforation and filling, 1Inf − 1 day after infection with HSV-1, and 1, 2, 3, 4, 8, 12, 16, 20, and 26 weeks after surgery. Data are presented as a dot plot, where “x” represents the mean healing score of the group with standard error of the mean bars. A Kruskal–Wallis nonparametric test for ordinal data followed by a Dunn’s post hoc test for multiple comparisons was performed; statistical details are in Supplementary Table 3. *Indicates statistical significance (*P* ≤ 0.05) between the treatment and control (cyanoacrylate glue) groups, **when *P* ≤ 0.01. The graphs’ red arrows indicate the time at which the CIS+SiNP-GF19+oint. group was treated with extra SiNP-GF19 in an ointment. *n* = 6 animals, except for cyanoacrylate group where at days 21 and 29, the group number decreased to 5 and 4 respectively, and the CIS+SiNP-GF19 group at day 21, when the animal number decreased to 5. **f** Control untreated eye and treated corneas of rabbits at 26 weeks after surgery. The surgical perforations were made in the center of each cornea, in the area that is hazy. Different degrees of haze can be observed as in-growing corneal stromal cells will scatter light, thereby causing the haze. In the cyanoacrylate group, the central patched area is opaque and red blood vessels can be seen entering the patch (neovascularisation).

With ethical permission from the Lithuanian State Food and Veterinary Service (permit # G2–179; 2021-05-28) and following the Association for Research in Vision and Ophthalmology guidelines, the left eyes of 24 male New Zealand white rabbits were operated to produce controlled, full-thickness corneal perforations that comprised 4 mm surface wounds extending 250 µm into the stroma, with 1 mm central perforations through the endothelial surface. As shown in Fig. 2, each perforation was filled with one of four different biomaterials: (1) cyanoacrylate adhesive, the standard of care control; (2) CIS; (3) CIS+SiNP-GF19; (4) CIS+SiNP-GF19+ointment (oint.). All CIS biomaterials gelled in situ within 5 min, forming firm gels after 20 min. Two days after surgery, all operated eyes were infected with 10^4^ plaque-forming units (PFU) of HSV-1 McKrae virus to mimic peri-surgical viral reactivation^22^. All animals showed photophobia, conjunctivitis, periorbital edema, and tearing, the clinical signs of inflammation resulting from perforation and HSV-1 infection (Fig. 3a–d). Cyanoacrylate-treated corneas possessed the highest inflammation scores for 6 weeks.

All treated corneas were assessed for inflammation and immune cell infiltration into the wound areas using an established scale^36^. The cyanoacrylate-treated group also had the highest level of inflammatory cell infiltration (Fig. 3e), with fibrosis and neovascularization (Fig. 3f) at the end of the 26-week (6-month) study period. The CIS group had the lowest inflammation and cell infiltration scores (Fig. 3a–e), followed by corneas treated with CIS+SiNP-GF19. In eyes given extended GF19 treatment (oint. group), inflammation was noted intermittently over 24 weeks (Fig. 3a–e). However, at the end of 26 weeks, all CIS-treated groups had relatively clear and non-vascularized corneas (Fig. 3f).

### Changes in inflammatory cytokine levels in rabbit tears

Surgical wounding and HSV-1 infection upregulated the production of pro-inflammatory cytokines, enzymes and other molecules (Supplementary Fig. 4, Supplementary Table 2)^37,38^. The levels of IL-1α, IL-1β and IL-17a fluctuated for all groups with no trend, suggesting that no auto-immune responses were observed.

The levels of pro-inflammatory IL-8, MIP1b, MMP9, NCAM-1, and TNF-α were high after the surgical perforations in all groups, most likely in response to wounding. In the cyanoacrylate group, IL-8, MIP1b, MMP9, NCAM-1, and TNF-α levels remained high after HSV-1 infection for about three weeks. Leptin levels remained low.

In the CIS-treated groups, IL-8, MIP1b, NCAM-1, and TNF-α levels were also elevated but lower than the cyanoacrylate group. After three weeks, all levels dropped. The initially high MMP9 levels decreased significantly within two days of surgery and remained low. Leptin levels were significantly higher in the cyanoacrylate group and remained high throughout the 26 weeks. At 3 weeks post-operation, a significant decrease in TNF-α was observed in the cyanoacrylate controls but not in the CIS groups. A significant increase in IL-21 was observed at weeks 3, 8, 20, and 26 postoperatively in the CIS+SiNP-GF19±oint. groups.

Similar changes in pro-inflammatory markers were observed in the untreated contralateral eyes although not as marked and levels were overall lower (Supplementary Fig. 5, Supplementary Table 3).

### Histopathological evaluation

**Fig. 4:**
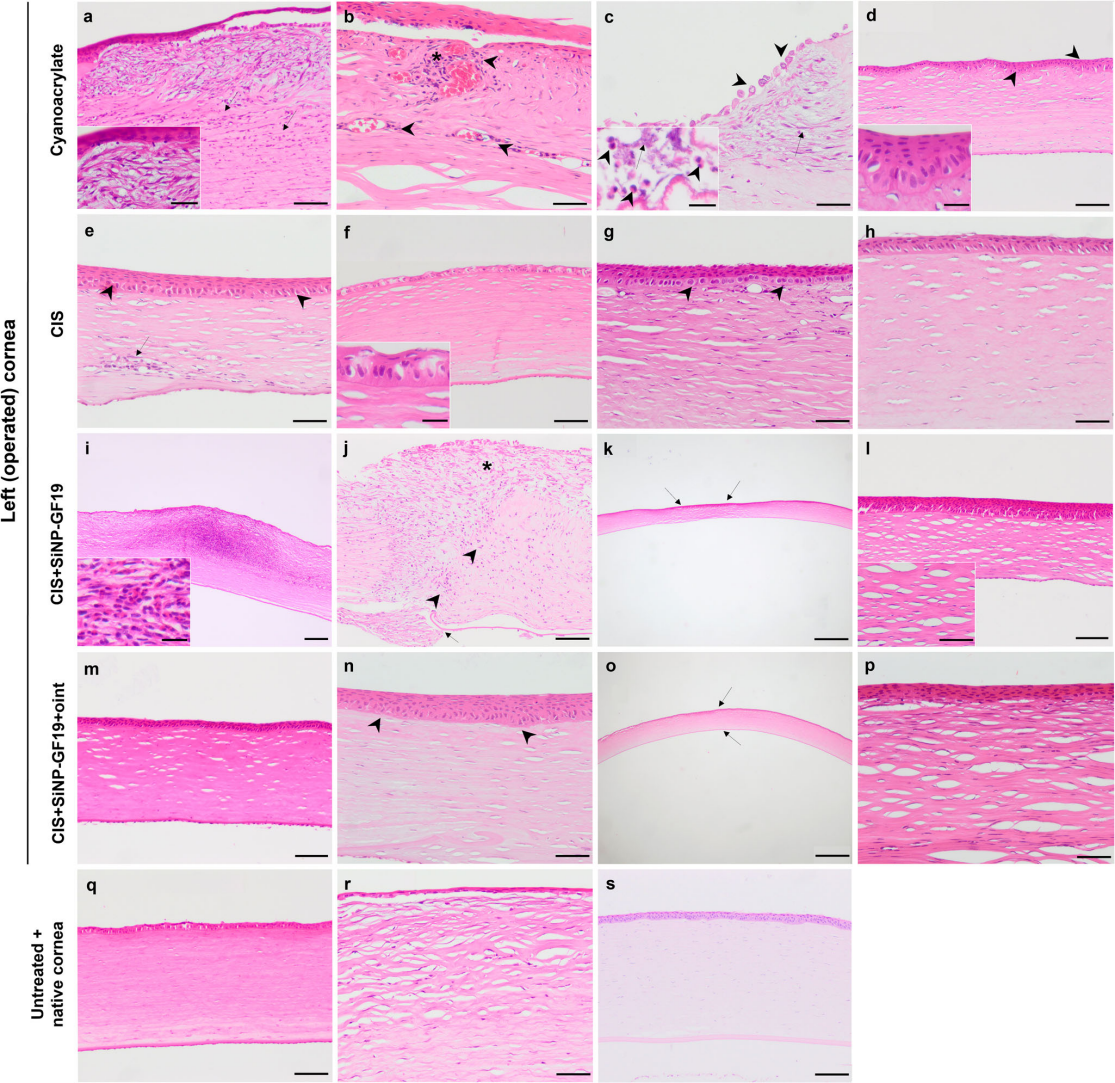
Hematoxylin & Eosin stained regenerated neocorneas at six months after treatment with various sealant-fillers, compared with untreated control corneas. Representative sections from cyanoacrylate-treated corneas showing **a** moderate, diffuse, mixed inflammatory infiltrate (arrows) and lipid keratopathy (inset), and **b** diffuse, mixed perivascular inflammatory infiltrate (asterisk) with neovascularization (arrowheads). **c** Cyanoacrylate-treated cornea showing lipid keratopathy (arrow), swollen epithelial cells (arrowheads), neutrophils (inset, arrowheads), and presence of bacteria (inset, arrow). **d** Epithelial hyperplasia (arrowheads and inset) and mild stromal disarrangement in a cyanoacrylate-treated cornea. **e** Regenerated neocorneas from the CIS-treated group showing focal inflammatory infiltrate (arrow) and epithelial hyperplasia (arrowheads); **f** vacuolar degeneration of the epithelium; **g** swelling of epithelial cells (arrowheads) and a moderate degree of stromal disarrangement; and **h** mild degree of stroma disarrangement and epithelial hyperplasia. **i** A sample from the CIS+SiNP-GF19 group showing severe keratitis with loss of the epithelium and Bowman’s membrane, with a severe mixed inflammatory infiltrate with free erythrocytes (hemorrhage) (inset). Other representative sections from the CIS+SiNP-GF19 group showing **j** severe disarrangement of all stromal layers with loss of epithelium (asterisk), rupture of Descemet’s membrane (arrow), and diffuse mild mixed inflammatory infiltrate (arrowheads); **k** stromal thinning (arrows); **l** mild to moderate stromal disarrangement and epithelial hyperplasia. Regenerated neocornea sections from the CIS+SiNP-GF19+oint. group showing **m** mild stromal disarrangement; **n** epithelial hyperplasia; **o** slight stromal thinning (arrows); and **p** moderate stromal disarrangement. **q** Representative sections of untreated cornea without significant histological changes. **r** An untreated cornea with severely disarranged layers within the stroma. **s** Representative microphotograph of a healthy native cornea. Scale bars in (**a**–**c**, **g**, **h**, **j**, **n**, **p**, **r**) represents 50 µm; (**d**–**f**, **l**, **m**, **q**, **s**) – 100 µm; (**i**) – 200 µm; (**k**, **o**) – 500 µm. Scale bars in inset from (**a**, **c**, **d**, **f**, **i**) represents 20 µm, and scale bar from (**l**) inset – 50 µm.

Results of histopathological evaluation by two veterinary pathologists are shown in Table 1 and Fig. 4. Corneas from animals that had died prematurely showed severe bacterial infection.

**Table 1:** Histopathological analyses of perforated and HSV-1 infected corneas receiving different treatments

Evaluation Criteria		*Cyanoacrylate*		*CIS*	*CIS* + *SiNP-GF19*	*CIS* + *SiNP-GF19+oint*.
Inflammatory infiltrate	I		25% [1/4] (diffuse mixed and perivascular) **50% [1/2] (mixed)**	-	**100% [1/1] (diffuse mixed)**	-
	II		**50% [1/2] (diffuse mixed)**	16.67% [1/6] (posterior focal mixed)	-	-
	III		-	-	20% [1/5] (focal mixed)	-
Neovascularization			75% [3/4], **50% [1/2]**	-	20% [1/5] (with hemorrhage)	-
Stromal disarrangement	I		50% [2/4], **50% [1/2]**	16.67% [1/6]	100% [5/5]	66.67% [4/6]
	II		25% [1/4]	16.67% [1/6]	-	16.67% [1/6]
	III		**50% [1/2]**	50% [3/6]	**100% [1/1]**	-
Lipid keratopathy			**100% [2/2]**	-	-	-
Stromal thinning			50% [2/4]	66.67% [4/6]	80% [4/5]	66.67% [4/6]
Epithelial hyperplasia			75% [3/4], **50% [1/2]**	66.67% [4/6]	80% [4/5]	66.67% [4/6]
Swelling of epithelial cells			50% [2/4], **100% [2/2]**	66.67% [4/6]	60% [3/5], **100% [1/1]**	-
Other findings			**50% [1/2]**^a^	16.67% [1/6]^b^	20% [1/5]^c^, **100% [1/1]**^d^	-

Results are expressed as the percentage and number of samples with the corresponding lesion out of the total samples examined for each experimental group (*n* = 6 samples/group). In cyanoacrylate and CIS+SiNP-GF19 groups, two and one rabbit, respectively, died prematurely, so the results from the left corneal examination from these animals are indicated separately in bold. Inflammatory infiltrate and stromal disarrangement are divided into 3 stages – I (mild), II (moderate) and III (severe)^36^.

^a^One of the left corneas from a cyanoacrylate treated animal which died prematurely showed severe septic keratitis with loss of the epithelium, necrosis, lipid keratopathy and presence of bacteria.

^b^One of the left corneas from CIS group showed vacuolar degeneration of epithelial cells.

^c^One of the samples from CIS+SiNP-GF19 showed severe keratitis with loss of the epithelium and Bowman’s membrane as well as severe mixed inflammatory infiltrate.

^d^Another sample from a CIS+SiNP-GF19 treated animal which died prematurely revealed severe disarrangement of all stromal layers with loss of epithelium and rupture of Descemet’s membrane, together with diffuse mild mixed inflammatory infiltrate.

Histopathological lesions were more frequent in the left treated and infected corneas from rabbits in the cyanoacrylate group than in the other groups (Fig. 4a–d). Whereas 50% (3 out of 6) animals from this group showed inflammatory infiltrates in the cornea (Fig. 4a, arrows, Fig. 4b, asterisk, and Fig. 4c, inset arrowheads), infiltrates were only observed in 16% (1 out of 6) and 33% (2 out of 6) of animals from the CIS and CIS+SiNP-GF19 groups, respectively. Only samples from cyanoacrylate and CIS+SiNP-GF19 groups displayed neovascularization; with the cyanoacrylate group showing a higher frequency in 66% (4 out of 6) of the animals (Fig. 4b, arrowheads). The CIS+SiNP-GF19+oint. group exhibited the lowest number of histological lesions, stromal disarrangement being the most frequent lesion in this group (Table 1; Fig. 4m, p). Interestingly, both animals that died prematurely from cyanoacrylate group had lipid keratopathy within their treated left corneas (Fig. 4a, inset, and Fig. 4c, arrow); this lesion was not found in any samples from the other groups. Epithelial hyperplasia (Fig. 4d), stromal thinning (Fig. 4o), and swelling of epithelial cells (Fig. 4g, arrowheads) were common histological findings in all four groups, except for the swelling that was not present in CIS+SiNP-GF19+oint. group.

No notable histological lesions were detected in the untreated corneas (Fig. 4q) of rabbits from all experimental groups except for one sample which showed severe disarranged layers within the stroma and moderate, diffuse, chronic infiltrate (Fig. 4r). Figure 4s shows a representative microphotograph of a healthy native cornea.

Histopathological examination of both right and left parotid and mandibular lymph nodes are summarized in Supplementary Fig. 6. Although lymphoid depletion was observed in specific samples, there were no detected differences in the degree of depletion among the different groups, nor between left and right nodes. Vascular alterations (diffuse congestion and parenchymal hemorrhage) in one left parotid lymph node from a CIS+SiNP-GF19 rabbit (Supplementary Fig. 6g), and two left mandibular lymph nodes from the CIS and CIS+SiNP-GF19 groups were noted.

### Regeneration of corneal tissue and nerves

**Fig. 5:**
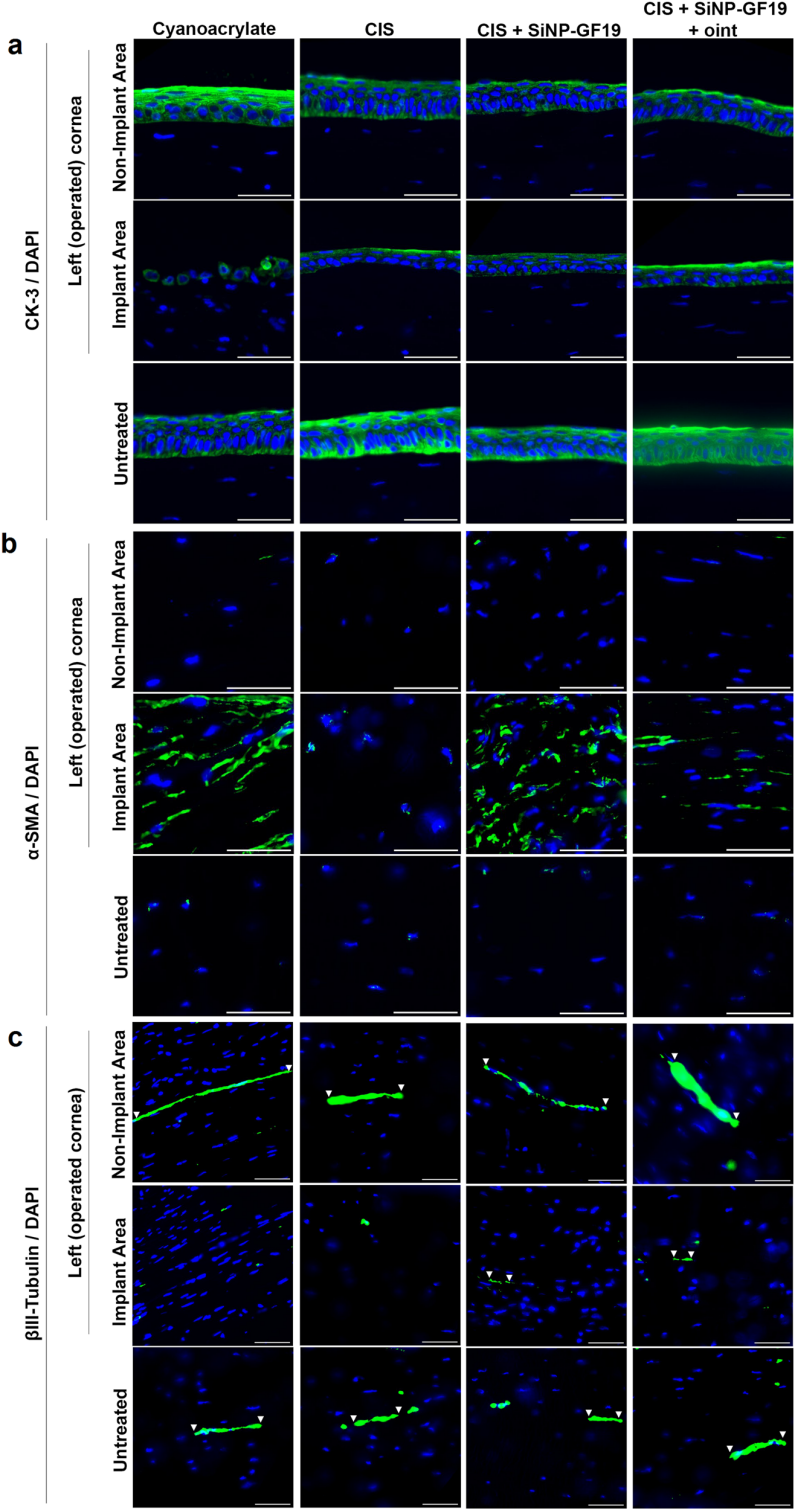
Regenerated rabbit neocorneas and adjacent areas in the different treatment groups, compared to untreated controls at six months post-operation. Representative sections from regenerated neocorneas after treatment with different sealant-fillers, showing fluorescence immunohistochemistry with antibodies against **a** cytokeratin-3 (CK-3; green); **b**, α smooth muscle actin (α-SMA; green); and **c** βIII tubulin (green). The white arrowheads indicate sections of nerves that were stained by the βIII tubulin antibody. In all images, nuclei were stained with DAPI (blue). Scale bars, 50 µm.

**Fig. 6:**
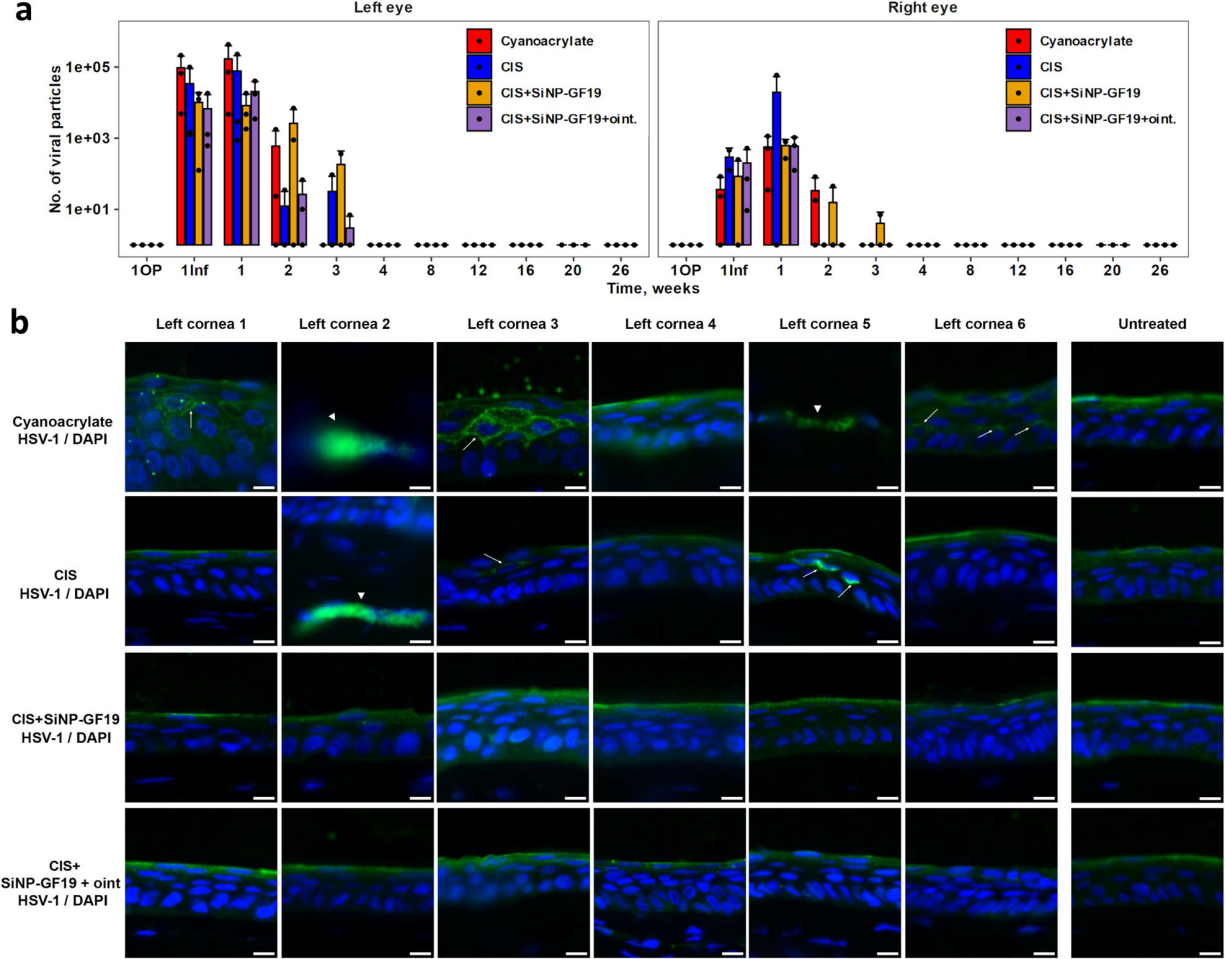
HSV-1 infection in rabbits. **a** Quantitative PCR analysis of HSV-1 DNA in rabbit tear samples. HSV-1 viral particles were detected in tear samples from the operated and virus-inoculated left eyes and untreated right eye at different time points: 1OP – 1 day after surgery; 1Inf – 1 day after infection (3 days after surgery); 1, 2, 3, 4, 8, 12, 16, 20 and 26 weeks after left eye surgery. Data are presented as group mean ± standard deviation. Points represent individual measurements. The red arrow in the graph indicates the time point at which the left eyes of the CIS+SiNP-GF19+oint. group animals started to receive anti-viral ointment. *n* = 3. **b** Representative images of regenerated neocorneas from HSV-1 infected and treated corneas at 6-month post-operation. Anti-HSV-1 staining (green) was performed to detect viruses within the tissues. The white arrowheads and arrows indicate HSV-1 staining in the anterior region of the stroma and the wing epithelial layer, respectively. In all images, nuclei were stained with DAPI (blue). Scale bars, 10 µm.

The regenerated epithelium of all corneas in the three CIS-treated groups were thinner than the adjacent untreated areas, but they nevertheless were positively labeled for cytokeratin 3 (CK-3), a marker of differentiated corneal epithelium^39^. In the cyanoacrylate group, however, the implant areas showed an absence of epithelium or a monolayer of unstructured CK3-negative epithelium (Fig. 5a). Staining in native corneas and negative controls are shown in Supplementary Fig. 7 for all the immunohistochemistry in Figs. 5 and 6.

Alpha-smooth muscle actin (α-SMA) expression is the hallmark of myofibroblasts^40^. Elevated α-SMA staining was observed in the implant area compared to non-implant areas and untreated contralateral corneas of the cyanoacrylate and CIS+SiNP-GF19 groups in particular. The CIS only group had the lowest α-SMA staining within the implant area (Fig. 5b). The untreated eye of one rabbit in the CIS group also showed enhanced α-SMA staining.

βIII tubulin expression is associated with corneal nerves and their regeneration^41^. Anti-βIII tubulin-stained corneal nerves were only found in implant areas of corneas treated with GF19-releasing CIS (Fig. 5c).

### HSV-1 infection in rabbits

Quantitative PCR analysis of HSV-1 DNA showed live virus in tear samples for 3 weeks indicating successfully induced peri-surgical infection in inoculated eyes (Fig. 6a). HSV-1 infection was found in the untreated contralateral eyes where the infection also lasted up to three weeks but was significantly milder, with ~100-fold lower number of virus particles recorded. At the end of six months, 5/6 operated eyes in the cyanoacrylate group and 3/6 of the CIS group revealed HSV-1 staining in the wing epithelial cells and/or the anterior stroma. There was no HSV-1 staining in the CIS+SiNP-GF19, CIS+SiNP-GF19+oint., and control untreated groups (Fig. 6b).

## Discussion

Our injectable CIS comprising CLP-LCPP and collagen-citrate glue gelled spontaneously, filling and sealing the perforated corneas and suppressing inflammation, demonstrating feasibility. With on-going HSV-1 infection, different degrees of regeneration were observed, depending on the presence or absence of GF19. Corneas treated with CIS releasing GF19 showed a higher level of inflammation compared to CIS alone, but were not as inflamed as the cyanoacrylate control group. However, very notably, CIS+SiNP-GF19+oint. treated regenerated neocorneas best approximated the morphology and function of a normal healthy cornea. This result confirms that inflammation is necessary for regeneration. However, the levels need to be controlled, as illustrated by the cyanoacrylate group, where uncontrolled inflammation resulted in structurally disorganized neocorneas.

In vitro infection studies showed that HSV-1 entered corneal cells resulting in cytopathic effects and cell death. When SiNP-GF19 was given prophylactically one hour prior to infection, it blocked HSV-1 entry as seen at 48 h post-infection. When SiNP-GF19 was given at the time of infection, the HCECs became infected but remained adherent and spread, suggesting that SiNP-GF19 conferred viability to infected cells. It has been shown that GF17, from which GF19 was derived, acts directly on viruses by disrupting their lipid membrane, and thereby preventing fusion to the cells for entry^27^. The parent LL37 peptide also acts directly on viruses^25^. GF19 most likely acts through a similar mechanism.

Cynanoacrylate adhesive is the main treatment used to seal emergency corneal perforations even though it is toxic and often needs multiple applications^6^. It causes eye irritation, inflammation, and kills adjacent cells thereby necessitating a follow-on corneal transplant^6^. All experimental CIS treatment strategies were significantly more effective at promoting repair and regeneration compared to the cyanoacrylate control. The cytokine-regulated response of corneas to all experimental treatment strategies were mostly similar and less acute than in the cyanoacrylate adhesive group. Significantly smaller scar tissues without ingrown vascular networks were formed in all the CIS treated groups, resulting in pupillary areas that were markedly optically clearer. Our results support the contention that MPC-containing hydrogels suppress inflammation and block neovascularization. This is in keeping with previous results in solid MPC-containing hydrogels tested in animals^18^ and in humans^17^.

The CIS+SiNP-GF19 and CIS+SiNP-GF19+oint. treated corneas showed increased conjunctival hyperaemia (red appearance), light sensitivity, tearing, and eyelid edema compared to the CIS only group. However, at six months post-operation, the CIS+SiNP-GF19+oint. group showed the most normal corneal morphology. We surmised that CLP-LCPP alone suppressed inflammation, while corneas treated with GF19 mounted an immune reaction against HSV-1 virus infection. The observed inflammation and immune infiltrates reflected changes in the expression of pro-inflammatory cytokines, MMP and other molecules.

All CIS groups had fully differentiated corneal epithelia, unlike the cyanoacrylate control group, showing that inflammation was suppressed sufficiently to allow for regeneration. Corneal epithelium often thickens to compensate for thinner stromas, accounting for the epithelial hyperplasia. Here, regenerated neostromas were likely thiner because the CIS contained over 95% water and most likely lost significant amounts of water through normal osmoregulation. Stromal myofibroblasts are essential for responding to infections and wounding but excessive numbers can cause fibrosis^40^. Myofibroblasts were present in cyanoacrylate-treated corneas as well as the CIS+SiNP-GF19 group, with much fewer cells in the CIS+SiNP-GF19+oint. group, mirroring the histopathology findings. βIII-tubulin antibody-stained corneal nerves were present in SiNP-GF19-treated corneas only suggesting that blocking of HSV-1 allowed faster regrowth of corneal nerves. LL37, the parent molecule of GF19, is reported to be a robust activator of immune responses^42^ including activation of pro-inflammatory cytokines and recruitment of immune cells to infection sites^43^. GF19 may function through similar mechanisms.

At weeks 2 and 3 post-treatment, CIS+SiNP-GF19 appeared to have more virus shedding than the CIS alone. However, the numbers were not significant. It is possible that while GF19 was released, it was only confined within the hydrogel patches while the HSV-1 infection could have spread to a region away from the patch and shed virus into the tears. The notable observation is that there was no viral reactivation over the 26-week observation period.

HSV-1 infection often involves the draining lymph nodes^44^. The histopathological analyses of the draining lymph nodes of treated and control eyes showed lymphoid depletion and presence of tingible body macrophages but no marked differences between treatment groups. Vascular alterations were observed in specific samples, but these were most likely incidental and unrelated to the corneal HSV-1 infection.

While active HSV-1 shedding lasted 3 weeks in all groups, anti-HSV-1 antibody staining revealed persistent HSV-1 infection or latency in corneal samples from the cyanoacrylate and CIS treatment groups only. The SiNP-GF19-treated corneas only showed background fluorescence equivalent to that seen in the control contralateral corneas. Interestingly, a significant increase in IL-21 was observed at week 3 postoperatively in the CIS+SiNP-GF19 treated group but not in the CIS+SiNP-GF19+oint. group. IL-21 is released during viral infection and helps control persistent infection^45^. LL37 upregulates the pro-inflammatory cytokine expression^46^, which likely includes IL-21. Therefore, GF19, which contains the bioactive anti-viral fragment of LL37, had most likely upregulated IL-21 to control the continuous HSV-1 infection. Hence, in HSV-1-free samples, there was no increased IL-21 production. Taken together with the in vitro data, SiNP-GF19 appears to have anti-HSV-1 activity, most likely in blocking viral entry into cells rather than being virucidal. The prolonged GF-19 treatment afforded by the ointment application allowed the regeneration of a more mature, less disorganized corneal stroma. The inclusion of a GF19 delivery system in CIS is an example of expanding the utility of the biomaterials for personalization of treatments, in this case, allowing for prophylactic prevention of peri-surgical HSV-1 reactivation (or infection).

In conclusion, our CIS sealed full-thickness perforations and suppressed inflammation in rabbit corneas while allowing regeneration. Our CIS could in the future be customized for specific patient needs, for example, to confer anti-viral activity to improve infected tissue regeneration as demonstrated by the delivery of GF19. Composite injectable biomaterials can, therefore, be designed to incorporate a nanoparticulate delivery system for drugs or bioactives needed for personalizing regenerative medicine-based therapies. Further iterations of optimization and testing can fine-tune them for future clinical applications.

## Methods

### CIS preparation and characterization

The CIS is an injectable hydrogel comprising two parts, a CLP-LCPP filler that promotes regeneration and a collagen-citrate glue. Each part was prepared in separate syringes that were shipped on dry ice and stored frozen at −80 °C until used.

#### LCPP synthesis and characterization

Heterobifunctional LCPP co-polymer (Fig. 1b) was synthesized using a one-pot method. Briefly, 885 mg of 2-methacryloyloxyethyl phosphorylcholine (MPC; Paramount Fine Chemicals, Dalian, China) was weighed and dissolved in 50 mL of distilled water. The solution was added to 150 mL of 0.5 M Tris-HCl, pH 6.7 in a 500 mL beaker, followed by 450 mg AC-PEG-COOH that was dissolved in the same beaker. The monomer solution was sonicated and nitrogen flushed for 30 min. Then, 51.3 mg of ammonium persulfate (APS, Sigma-Aldrich, Oakville, ON, Canada) was added. The solution was re-sonicated and nitrogen flushed for 5 min, after which 33.6 µL of tetramethylethylenediamine (TEMED, Sigma) was mixed into the solution. The solution was split into 3 equal portions into three 100 mL round bottom flasks in a Carousel chemical reactor (Heidolph™ Radleys Carousel 6 Plus Reaction Station™, Heidolph North America, Wood Dale, IL, USA) and allowed to react for 24 h under positive pressure and nitrogen atmosphere with constant stirring at 20 °C. After the reaction was completed, the solution was dialyzed for 48 h using cellulose dialysis membrane (Spectra/Por 4 Dialysis Tubing, 12–14 kDa MWCO) in a single pass against 50 L of ddH_2_O. The dialysate was then lyophilized and stored at −20 °C until used.

Characterization of LCPP was done using proton nuclear magnetic resonance spectroscopy (^1^H NMR and ^31^P NMR) in D_2_O on a Bruker, Avance III HD 600 MHz NMR Spectrometer (Bruker, Billerica, MA, USA), Fourier-transform Infrared spectroscopy (FTIR-ATR) (Thermo Scientific, Nicolet 6700 / Smart iTR, Waltham, MA, USA) was used to characterize polymers. Differential Scanning Calorimeter (DSC 25, TA Instruments – Waters LLC, New Castle, DE, USA) was used for thermal analysis. Each sample was placed inside the sealed aluminum-lead pans and subjected to a scanning rate of 10 °C/min up to 150 °C.

#### CLP-LCPP and collagen-citrate components

CLP with the sequence (DOG) _4_(POG) _4_(PKG) _4_^47^ was synthesized by GenScript Biotech Corp. (Piscataway, NJ). Aqueous stock solutions of the following were prepared: 15% w/w CLP stock, heated to 65 °C to dissolve; 10% LCPP stock solution; 10% (4-(4,6-dimethoxy-1,3,5-triazin-2-yl)−4-methyl-morpholinium chloride) (DMTMM, Sigma-Aldrich). For CLP-LCPP preparation, 10 µL of LCPP stock and 7.5 µL of DMTMM stock were added to 32.5 µL of MOPS at 65 °C. The solution was allowed to react for 1 min. A tube of 50 µL of the CLP stock was prepared at 65 °C. The two tubes were combined and mixed by vortexing for 30 s. Then, 100 µL of the solution was added to a syringe (1 mL tuberculin slip tips). The syringe was kept on dry ice for the entire procedure and immediately covered (within 10 s) after filling to freeze the solution and stop the reaction from progressing. The syringe was stored frozen at −80 °C until used. For preparation of collagen-citrate glue, a 20% w/w collagen stock was prepared by dissolving porcine collagen type 1 (Nippon Ham, Nippon Meat Packers, Japan) in MOPS (0.5 M pH 6.8) at 60 °C. Also, 10% citric acid (Sigma-Aldrich) stock was prepared in MOPS; then, 7.2 mg of DMTMM was dissolved in 15.4 µL of citric acid stock. The solution was allowed to react for 1 min. The DMTMM/citric acid solution was added to 40 µL of collagen stock solution and mixed by pipetting. The solution was allowed to react for 30 s, and 50 µL was then pipetted to a syringe (1 mL tuberculin slip tips). All CLP-LCPP and collagen-citrate glue syringes were filled on dry ice, stored frozen and shipped at −80 °C until used.

#### CIS characterization

The light transmission of hydrogels between 200 and 1000 nm was evaluated by placing the hydrogel inside the wall of a quartz cuvette filled with PBS and reading its absorption in a Spark multimode microplate reader (TECAN NanoQuant Plate^TM^ Switzerland). A cuvette filled with only PBS was used as the baseline reference. Measured absorbance was then converted to the corresponding percent of transmission.

The water content of hydrogels was evaluated by weighing the “dry weight” (W_0_) of the samples and then comparing this to the weight of the material after being soaked in water at room temperature until a constant weight was achieved (W). The total water content of the hydrogels (Wt) was calculated according to Eq. (1) below:

1 $${W}_{t}=\frac{(W-{W}_{0)}}{W}\times 100.$$

The experiments were done at least 3 times and the results were reported as an average.

#### Bursting pressure evaluation

Excised pigs’ eyes were obtained from a local butcher shop. The corneas with a scleral rim were dissected out and fixed in place using an artificial anterior chamber (Barron Artificial Anterior Chamber, Katena, NJ, USA). A corneal button of 5 mm diameter to 50% depth was removed using a trephine and lamellar dissection, followed by a 1 mm central full-thickness perforation made using a biopsy punch. To seal the prepared perforation, various formulations were then applied. After three hours, the bursting pressure was measured to confirm that the hydrogel inside the perforation had fully crosslinked.

### HSV-1 viral stock and titers

The HSV-1 McKrae strain, a gift from D.J. Carr, Univ. of Oklahoma Heath Sciences Center, OK, USA, was grown in Vero cells (ATCC, CCL-81) supplemented using DMEM-Hi glucose (Sigma-Aldrich, St. Louis, MO, USA) with 10% FBS (Wisent, St-Bruno, QC, Canada) and penicillin-streptomycin (Gibco, Thermo Fisher Scientific, Waltham, MA, USA). Once the cells were close to confluence, the media was replaced by serum-free media containing serial dilutions of viruses. Cells were incubated with the virus for 1 h, shaking every 15 min, after which the virus was removed. The viral suspension was removed, and the cells were rinsed with PBS. Viral concentrations were determined using the liquid overlay method^48^ using 1.2% Avicel® PH-101 (Sigma-Aldrich) in DMEM-Hi glucose. After 3 days, the liquid overlay was removed, and cells were fixed with 10% formaldehyde for 30 min. Then, 0.5% crystal violet (Sigma-Aldrich) was used to stain the cells and count the plaques.

### Preparation and characterization of silica nanoparticle loaded GF19

#### GF19 peptide synthesis

Fmoc protected amino acids and low-loading Wang resin were purchased from CEM Corporation (Matthews, NC, USA). All peptides were synthesized using microwave assisted Fmoc solid phase peptide synthesis using a Liberty Blue automated system (CEM Corp.). Briefly, the required amount of resin was swelled in DMF for 5 min. Next, Fmoc deprotection was carried out with 20% piperidine at 90 °C for 60 s. Standard coupling cycles using DIC/Oxyma Pure were run at 90 °C for 240 s in each amino acid. Peptides were cleaved from the resin and deprotected with TFA/TIS/EDT/H_2_O (92.5/2.5/2.5/2.5% v/v) at 42 °C for 30 min, and then precipitated in –20 °C diethyl ether. Peptide crude products were then dried under vacuum overnight and purified by RP-HLPC in a Waters 1525EF semi-preparative system (Waters Corp. Milford, MA, USA) with a 21.6 × 250 mm C18 column at 20 mL/min. Peptide purity and identity was confirmed via RP-UPLC-UV/MS in a Waters Acquity UPLC Xevo TQD (Waters Corp. Milford, MA, USA), using a 2.1 × 100 mm UPLC BEH C8 column. A purity of >95% was determined through HPLC peak analysis. The theorical molecular ions found for each peptide is described on Supplementary Tables 3 and 4, and Supplementary Fig. 4 in Supplementary Materials.

#### Loading of GF19 in silica nanoparticles

GF19 was loaded in solid silica nanoparticles (SiNPs) as we previously described for its parent peptide, LL37^33^. Briefly, 6.7 mL of Triton X-100 (Sigma-Aldrich, Oakville, ON, Canada) was dissolved in 20 mL of cyclohexane (VWR International, Mississauga, ON, Canada) in a round-bottomed flask immersed in oil bath at 50 °C. Then, 8 mg of GF19 was dissolved in 2 mL distilled water and mixed with cyclohexane-Triton X-100 solution to generate reverse w/o microemulsion. Next, 2.5 mL of TEOS (VWR International, Mississauga, ON, Canada) was dropped slowly followed by 0.33 mL of ammonium hydroxide solution. The reactants were stirred for 48 h at 50 °C. The as prepared SiNPs were washed with ethanol (ChapTec, Montreal, QC, Canada) thrice followed by distilled water and then lyophilized for storage. They were reconstituted with PBS for further use.

#### Characterization of SiNP-GF19

The zeta potential and particle size of the prepared samples were measured on a Zeta View Z-NTA nanoparticle tracking analyzer (Particle Metrix PMX 120-12C-R4, Ammersee, Germany). Each SiNP and SiNP-GF19 was diluted using DW to form a 1:1000 W/W dispersion; then, the sample was injected to Zeta view instrument to determine particle size and zeta potential. Thermogravimetric analysis was performed on a Thermogravimetric Analyzer (TGA) Q500 (TA Instruments – Waters LLC, New Castle, DE, USA) to determine the composition of SiNP-GF19. 30 mg of each dried sample was placed inside the TGA instrument and heated up to 730 °C under nitrogen gas at the rate of 10 °C per min^34^. The X-ray diffraction patterns were recorded by an X-ray diffractometer (XRD) (D8 Advance, Bruker) using monochromatic Cu Kα1 radiation. Differential Scanning Calorimeter (DSC 25, TA Instruments) thermograms were recorded by placing each sample (∼3 mg) inside sealed aluminum-lead pans and heating at a scanning rate of 10 °C/min up to 400 °C.

#### SiNP-GF19 encapsulation efficiency and release study

For the calculation of EE of prepared GF19-loaded SiNPs, GF19 was tagged by FITC to be monitored easily by a UV-Visible spectrophotometer. The following equation, Eq. (2), was used for the evaluation of EE (%):

2 $${EE}\,( \% )=\frac{{Weight}\,{of}\,{drug}\,{in}\,{SiNPs}\,}{{Initial}\,{weight}\,{of}\,{drug}}$$

For this purpose, 10 mg freeze-dried loaded NPs were dissolved in 1 mL of PBS, and after 24 h, the drug content was determined using a UV–visible spectrophotometer (TECAN NanoQuant Plate^TM^) at a wavelength of 454 nm.

For the drug release profile, a certain amount of GF19-loaded SiNPs powder was dispersed in 2 mL PBS into a dialysis sac and immersed into 30 mL of PBS at pH 7.4 at 35 °C, in an air bath shaker. At different time intervals, 1 mL of solution was withdrawn from the main vial and exchanged with 1 mL of fresh PBS. The concentration of GF19 was measured by UV–Visible spectrophotometer at 454 nm.

### Composite CIS with SiNP-GF19

#### CIS incorporating SiNP-GF19

Preparation of CLP-LCPP with GF19 carrying NPs was performed in a corresponding manner to the gels lacking nanoparticles. Briefly, 15 µL of LCPP stock and 11.3 µL of DMTMM stock was added to 33.7 µL of 3-morpholinopropane-1-sulfonic acid (MOPS) buffer at 65 °C. The solution was allowed to react for 1 min. Then, 15 µL of 10X nanoparticle solution (loaded with GF19) was added to 75 µL of CLP stock. The two tubes were combined and mixed by vortexing for 30 s. Then, 100 µL of the solution was aliquoted to a syringe (1 mL tuberculin slip tips). The syringe was kept on dry ice for the entire procedure and immediately covered after filling to freeze the solution and stop the reaction from progressing. The syringe was stored frozen at −80 °C until shipped for use. The collagen-citrate glue here was made using the same method as the hydrogel without SiNP.

#### GF19 in vitro biocompatibility

Immortalized human corneal epithelial cells (HCECs)^32^ were cultured in 48-well plates in humidified incubator at 37 °C and 5% CO_2_. The growth media used was KeratinoMax, serum-free media for keratinocytes along with KeratinoMax supplement (Wisent, St-Bruno, QC, Canada). Once HCECs were 90–95% confluent, they were treated by SiNP, SiNP-GF19, and CIS with SiNP-GF19. Cultures were evaluated at 48 h, 72 h, and 96 h.

#### GF19 in vitro anti-viral efficacy

HCECs were grown overnight in 12-well chambered slides (Ibidi GmbH, Gräfelfing, Germany). They were incubated with SiNPs loaded with 5 µM FITC-GF19 for one hour and then infected with HSV-1 McKrae virus at a MOI of 0.5. After one hour of virus exposure, the media was replaced with fresh media (without virus or peptide). The cells were left to grow for 48 h, after which they were fixed in 4% paraformaldehyde in 0.1 M PBS for immunocytochemistry. A polyclonal anti-HSV-1 antibody raised in goat (1/1000 dilution; FisherSci Cat. #PA17493, Thermo Fisher Scientific, Waltham, MA, USA) and a donkey anti-goat IgG secondary antibody conjugated with DyLight™ 550 (1/500; Invitrogen Cat. #SA510087, Thermo Fisher, Burlington, ON Canada) were used to visualize virus particles. Imaging was performed on Zeiss Confocal LS M880 upright multiphoton system (Zeiss, Oberkochen, Germany) with analysis performed using FIJI software (Image J2, version 2.3.0/1.53q)^49^. Controls comprised uninfected cells treated with SiNP-GF19 and untreated HSV-1 infected cells.

### In vivo rabbit surgical perforation and HSV-1 infection model

The study design is summarized in Fig. 2. Ethical permission was obtained from the Lithuanian State Food and Veterinary Service, No. G2-179; 2021-05-28. HSV-1 infects both males and females equally^50^. For the study 24 male New Zealand white rabbits were studied as they were more docile for eye examinations. The rabbits were randomly divided into four groups in order to test different sealant-fillers. Following the guidelines of the Association for Research in Vision and Ophthalmology (ARVO), the left eyes of 24 rabbits were operated to produce controlled corneal perforations. Before surgery, each animal was anaesthetized by an intramuscular injection of 35 mg/kg Bioketan 100 mg/ml (Vetoquinol, Poland) and 5 mg/kg Sedaxylan 20 mg/ml (Eurovet, Netherlands). Just prior to the surgery, one drop of 5% Betadine (Alcon, Belgium) was applied to the left eye of the animal to disinfect the ocular surface. During the surgery, one drop of local anesthetic, Alcaini 0.5% (Alcon-Couvreur, Belgium) was applied every 10 min for pain control. After filling and sealing the surgical perforations in each treated cornea, one more drop of 5% Betadine was applied. Each eye was then given Tobrex ointment (Alcon-Couvreur, Belgium) 15 min later, and thereafter, ointment was applied once a day for the next 3 weeks. In addition, for 5 days after the surgery, pain was managed by subcutaneous injection of 4.0 mg/kg Rycarfa 50 mg/ml (KRKA; Slovenia).

Each surgical perforation was performed in two stages: (i) a 4 mm trephine was used to make a 250 μm deep circular incision, and a sublaminar excision of the corneal stroma was performed; (ii) a 1 mm trephine was then used to make a perforation of the cornea at the center of the central part. The wound bed of each cornea was filled and sealed using one of: (1) cyanoacrylate adhesive (control group); (2) CIS; (3) CIS+SiNP-GF19; (4) CIS+SiNP-GF19+oint. from weeks 3 to 6 week postoperatively. The right, contralateral corneas served as untreated controls.

To fill perforations, CIS syringes (CLP-LCPP and collagen-citrate) ± SiNP-GF19 were heated at 65 °C for 5 min before use. Collagen-citrate glue was applied on the wound bed, and then CLP-CLP filler was added, and then allowed to gel in situ. Each operated eye was treated with Tobrex® (ophthalmic ointment containing 0.3% tobramycin) once a day (Tobrex, Novartis, East Hanover, NJ) each day for the first 3 weeks post-operation. Two days after surgery, the left eyes of rabbits in all groups were each infected with 1 × 10^4^ PFU of HSV-1 virus, McKrae strain, to mimic a peri-surgical viral reactivation. At three weeks post-operation, the CIS+SiNP-GF19+oint. group were treated with SiNP-GF19 ointment for a further three weeks. Corneal healing was followed over 26 weeks (6 months).

At the end of the experiment, rabbits were anesthetized with 35 mg/kg Bioketan 100 mg/ml (Vetoquinol, Poland) and 5 mg/kg Sedaxylan 20 mg/ml (Eurovet, Netherlands) by intramuscular injection. They were then euthanized with a 5 ml injection of Exagon 400 mg/ml (Richter Pharma AG, Austria) by intracardic injection to the animal.

### Clinical examinations of rabbit eyes

All operated and infected eyes and their contralateral controls were examined for photophobia, conjunctival injection, serous secretions, and eyelid edema. If eyes were healthy, their eyes received a score of 0, if mild pathologies were observed – a score of 1, and if pronounced clinical symptoms were seen a score of 2. Furthermore, wound healing was also tracked by evaluating progressive cornea epithelial and stroma infiltration^36^. The following scale was used: 0, transparent; 1, slight opacity, partially or completely covering the pupil; 2, non-intense opacity covering the anterior segment of the eye; 3, intense opacity, partially or completely covering the pupil; and 4 intense opacity, covering the anterior segment and the corneal perforation. Clinical examinations of rabbit eyes were performed periodically by 2–3 independent researchers and a mean score of each animal evaluation was registered throughout 26 weeks after the surgery. Photophobia, conjunctival injection, serous secretions, and eyelid edema data are presented as heat maps, where each square represents the average of ordinal data of the corresponding group, whereas progressive cornea epithelial and stroma infiltration data is presented as a dot plot, where each dot represents the individual animal score.

#### Collection of tear samples

Tear samples from both eyes of each rabbit were collected at the following time points: the day after surgery (1OP), the day after infection (1Inf), and at weeks 1 to 4, 8, 12, 16, 20, and 26. For this purpose, Schirmer tear test strips (Haag-Streit UK Ltd, Harlow, UK) were placed in the inferior anterior conjunctiva of each eye and left until the strip was wet to 2 cm. The collected tear samples (in strips) were stored in sterile Eppendorf tubes at −80 °C until use.

#### Estimation of HSV-1 DNA content by real-time quantitative PCR analysis (qPCR)

For DNA isolation, strips of tear samples from the left eye were cut at the 1.8 cm mark and ground into 2 mm long pieces. 250 μL of 0.01 M phosphate buffer saline (PBS; Thermo Fisher Scientific) was added to the crushed strips. Next, the samples were heated for 15 min. At 72 °C for 15 min, followed by incubation at room temperature for a further 4 h. After incubation, the samples were centrifuged for 1 min at 10,000 rpm and the resulting supernatant was collected. The GeneJET Viral DNA/RNA Purification Kit (Thermo Fisher Scientific) was used for DNA isolation and all procedures were performed according to the manufacturer’s recommendations. 30 µL of 2 × 10^8^ PFU/mL HSV-1 solution was used for the calibration curve. An unused Schirmer tear test strip was used as negative control. PCR was performed using a TaqMan® Universal PCR Master Mix (Life Technologies, Carlsbad, CA, USA). All procedures were performed according to the manufacturer’s recommendations. The primers used were 5′-CCTTTCTCCA-GTGCTACCTG-3′ and 5′-GCCAGAATGACAAA CACGAAG-3′. The fluorescent probe sequence was 5′−6FAM/TG TCC TTA A/Zen/T GTC CGC CAG ACG C/3IABkFQ-3′. qPCR reactions and analysis were performed using a QuantStudio™ 3 Real-Time PCR system (Thermo Fisher Scientific, Waltham, MA, USA). Reaction protocol: 95 °C for 3 min; [(95 °C for 30 s) + (61 °C for 1 min)] x 45 cycles.

#### Cytokine variation assay using ELISA arrays

A Rabbit Cytokine Antibody ELISA Array (Abcam, Cambridge, United Kingdom) was used to analyze the changes in the expression profiles of 10 cytokines (IL-1α, IL-1β, IL-8, IL-17a, IL-21, Leptin, MIP-1b, MMP-9, NCAM-1, TNF-α). Randomly selected tear samples from both eyes of three rabbits per group collected throughout the experiment were used. The tear strips of the three rabbits were pooled into a single sample for a single group, single time point analysis. The tear strips were prepared for analysis by cutting the tear strip at the 1.8 cm mark and immersing it in NaCl saline solution (Fresenius Kabi AB, Bad Homburg, Germany). The samples were incubated for 3 h. At 37 °C with shaking at 300 rpm followed by centrifugation at 10,000 rpm for 1 min and collection of the supernatant. The analysis of the samples obtained was carried out according to the recommendations of the ELISA array manufacturer. The grids were measured and analyzed on an InnoScan 710 microarray scanner system (Innopsys, Carbonne, France).

### Histopathological examination of corneas and lymph nodes

The implant area of each cornea from both eyes as well as parotid and mandibular lymph nodes, right and left, were routinely processed, and embedded in paraffin. Four-micron sections were stained with hematoxylin-eosin (H&E) and blindly evaluated by two veterinary pathologists. For cornea samples, three non-consecutive sections were examined. In all samples, the presence of stromal inflammatory infiltrate, neovascularization, stromal disarrangement, lipid keratopathy, epithelial hyperplasia, and swelling of epithelial cells were evaluated. The stromal inflammatory infiltrate and the stromal disarrangement were scored, being I, mild, II, moderate, and III, severe. Other findings, such as the presence of bacteria, vacuolar degeneration of epithelial cells, or vascular alterations were also evaluated. For parotid and mandibular lymph nodes, the level of lymphoid depletion was scored as follows: 0, no microscopic changes (<10% of the tissue was affected); I, mild to moderate microscopic changes (from 10–50% of the tissue was affected); II, severe microscopic changes (>50% of the tissue was affected). Likewise, the presence of tingible body macrophages on the tissue was scored from 0 to II. The presence of germinal center activation, vascular changes, inflammatory cells, and mitotic figures was also recorded.

### Fluorescence immunohistochemistry

The expression of CK-3, βIII tubulin, α-SMA, and HSV-1 was determined by fluorescence immunohistochemistry, using 4% formaldehyde-fixed paraffin-embedded tissue samples. After paraffin embedding, sections at 5 μm thickness were first deparaffinized in xylene and rehydrated through a graded series of alcohols (100%, 95%, 70%, 50%, and water). Next, tissue sections were incubated in Tris-EDTA buffer (10 mM Tris base, 1 mM EDTA solution, 0.05% Tween 20, pH 9.0) at 97 °C for 25 min in a water bath for antigen retrieval. The sections were then permeabilized in Tris-buffered saline (TBS) plus 0.025% Triton X-100, and blocked with TBS containing 10% fetal bovine serum (FBS) and 1% bovine serum albumin (BSA). Next, tissue samples were exposed to the following primary antibodies: (i) mouse anti-CK-3 monoclonal antibody (1/50; Abcam Cat. #ab68260, Cambridge, UK); (ii) mouse anti-α-SMA monoclonal antibody (1/800; Invitrogen Cat. #MA5–11547, Waltham, MA, USA); (iii) mouse anti-βIII tubulin monoclonal antibody (1/500; Invitrogen Cat. #MA1-118); (iv) goat anti-HSV type 1 polyclonal antibody (1/1000; Invitrogen Cat. #PA1-7493). After an overnight incubation at 4 °C in humidifying conditions, an anti-mouse Alexa488-conjugated secondary antibody (1 μg/mL; Invitrogen Cat. #A-11001) for CK-3, βIII tubulin, and α-SMA, and an anti-goat Alexa488-conjugated secondary antibody (5 μg/mL; Invitrogen Cat. #110055) for HSV-1 was added for 1 h at room temperature. Finally, the slides were stained with 1 μg/mL DAPI (Sigma; San Louis, MO, USA) and mounted in a fluorescence mounting medium (Agilent; Santa Clara, CA, USA). Images were taken on an inverted fluorescence microscope (Leica Microsystems, Barcelona, Spain). Negative control samples without the primary antibody were included to assess non-specific staining. Native rabbit corneas were used as controls.

### Statistical analyses

R statistical software (4.1.2, https://www.r-project.org/) was used for statistical analysis of all data and plotting of graphs. For the analysis of ordinal data, the Kruskal-Wallis test for non-parametric statistics was used, and differences between groups were compared with Dunn’s post hoc test. Tear cytokine ELISA data were treated as normally distributed and two-factor ANOVA was used for analysis. Differences between groups were compared with Tukey HSD post hoc test. Data were visualized with different types of graphs as indicated in the respective figure legends. One-way ANOVA with Tukey post hoc test was used for fluorescence intensity (MFI) of HSV-1 staining. Mean fluorescence intensity (MFI) of HSV-1 staining in virus-infected untreated *vs*. SiNP-GF19-treated cells were evaluated by Student’s test. *P* ≤ 0.05 was considered statistically significant in all the tests

### Reporting summary

Further information on research design is available in the Nature Research Reporting Summary linked to this article.

### Supplementary information

Supplementary material of Simoliunas et al

Reporting summary

## Data Availability

The datasets generated during the current study are available in the Figshare repository (https://figshare.com/s/e112efc30dfa0b17e954). The image datasets generated during the current study are available from the corresponding author on reasonable request.

## Code availability

R statistical software, version 4.1.2 was used for statistical analysis of all data and plotting of graphs. Open source Fiji software was used for quantitation of Mean Fluorescence Intensity (MFI) - (ImageJ2 - version 2.3.0/1.53q, USA; https://imagej.net/software/fiji/).
