# Supplementary material for: Inflammation-suppressing cornea-in-a-syringe with anti-viral GF19 peptide promotes regeneration in HSV-1 infected rabbit corneas
Source: NPJ Regen Med. 2024 Mar 1;9:11. doi: 10.1038/s41536-024-00355-1 (PMC10907611; doi:10.1038/s41536-024-00355-1)
Supplement: Supplementary file 1 — Supplementary material of Simoliunas et al [file 41536_2024_355_MOESM1_ESM.pdf]

Supplementary Information

Inflammation-Suppressing Cornea-in-a-Syringe with Anti-viral GF19 Peptide Promotes Regeneration in HSV-1 Infected Rabbit Corneas

Egidijus Simoliunas[1,†], Inés Ruedas-Torres[2,†], Yolanda Jiménez-Gómez[3,†], Elle Edin[4,5,†], Mozhgan Aghajanzadeh-Kiyaseh[4,5], Mostafa Zamani-Roudbaraki[4,5], Rimvydas Asoklis[6], Milda Alksne[1], Neethi C. Thathapudi[4,5], Bijay K. Poudel[4,5], Ieva Rinkunaite[1], Kasparas Asoklis[1], Monika Iesmantaite[1], Laura Ortega-Llamas[3], Almantas Makselis[6], Marcelo Munoz[7], Daiva Baltriukiene[1], Virginija Bukelskiene[1,*], Jaime Gómez-Laguna[2,*], Miguel González-Andrades[3,*], May Griffith[4,5,*]

[1]Department of Biological Models, Institute of Biochemistry, Life Sciences Center, Vilnius University, Lithuania
[2] Department of Anatomy and Comparative Pathology and Toxicology, Pathology and Immunology Group (UCO-PIG), UIC Zoonosis y Enfermedades Emergentes ENZOEM, University of Córdoba, International Excellence Agrifood Campus 'CeiA3', 14014 Córdoba, Spain
[3] Maimonides Biomedical Research Institute of Cordoba (IMIBIC), Department of Ophthalmology, Reina Sofia University Hospital and University of Cordoba, 14004 Cordoba, Spain.
[4]Department of Ophthalmology and Institute of Biomedical Engineering, University of Montreal, Montreal, Quebec, Canada
[5]Maisonneuve-Rosemont Hospital Research Centre, Montreal, Quebec, Canada
[6]Department of Ophthalmology, Vilnius University Hospital, Lithuania
[7]University of Ottawa Heart Institute, Ottawa, Ontario, Canada

[†]These authors contributed equally to this work
[*]Corresponding author

Supplementary Tables

Supplementary Table 1. Statistical output from the assessment of the healing process in the left operated eye over 26 weeks as shown in Fig. 3e. Statistical assessments were performed using the Kruskal-Wallis nonparametric test for ordinal data with the Dunn's post hoc test for multiple comparisons. n=6 animals, except for cyanoacrylate group where at days 21 and 29 post-treatment, the number decreased to 5 and 4 respectively, and the CIS+ SiNP-GF19 group, where the animal number decreased to 5 at day 21.

Time point	Kruskal-Wallis test	P value	n
1Inf	X2(3) = 8.44	0.038	23
3 weeks	X2(3) = 15.02	0.0018	22
4 weeks	X2(3) = 10.02	0.018	21

Supplementary Table 2. Statistical output of the analyses of the temporal variation in the concentration of inflammatory cytokines in the tears of eyes surgically perforated and HSV-1 infected corneas shown in Supplementary Fig. 4. n=4 technical repeats of 3 pooled samples from each group. The data was assessed using two-way ANOVA with the Tukey HSD post hoc test for multiple comparisons.

Protein	Two-way ANOVA test	P value	Eta-squared (η^2)
IL-1a	F(30,124) = 25.42	9.43×10^{-40}	0.860
IL-1b	F(30,132) = 3.77	7.06×10^{-8}	0.462
IL-8	F(30,129) = 175.66	2.03×10^{-90}	0.976
IL-17a	F(30,132) = 0.79	0.77	0.152
IL-21	F(30,120) = 9.15	3.04×10^{-19}	0.696
Leptin	F(30,105) = 13.78	1.55×10^{-24}	0.797
MIP1b	F(30,130) = 68.73	9.6×10^{-66}	0.941
MMP9	F(30,128) = 134.07	1.34×10^{-82}	0.969
NCAM-1	F(30,115) = 27.24	2.45×10^{-39}	0.877
TNFa	F(30,110) = 20.97	5.31×10^{-33}	0.851

Supplementary Table 3. Statistical output of the analyses of the temporal variation in the concentration of inflammatory cytokines in the tears of non-treated contralateral eyes of rabbits that are shown in Supplementary Fig. 5. n=4 technical repeats of 3 pooled samples from each group. The data was assessed using two-way ANOVA with the Tukey HSD post hoc test for multiple comparisons.

Protein	Two-way ANOVA test	P value	Eta-squared (η^2)
IL-1a	F(30,128) = 16.91	1.39×10^{-31}	0.798
IL-1b	F(30,132) = 3.14	3.62×10^{-6}	0.416
IL-8	F(30,132) = 194.99	5.4×10^{-95}	0.978
IL-17a	F(30,131) = 1.51	0.059	0.257
IL-21	F(30,120) = 10.68	7.56×10^{-22}	0.728
Leptin	F(30,110) = 11.08	1.78×10^{-21}	0.751
MIP1b	F(30,132) = 9.27	2.37×10^{-20}	0.678
MMP9	F(30,130) = 66.57	6.57×10^{-65}	0.939
NCAM-1	F(30,115) = 11.04	6.31×10^{-22}	0.742
TNFa	F(30,112) = 26.248	6.7×10^{-38}	0.875

Supplementary Table 4. Peptide synthesis reference of LL37, GF17, GF19, and GF19-FITC synthesized for this work.

Peptide Name	Sequence
LL37 (Reference)	LLGDFFRKSKEKIGKEFKRIVQRIKDFLRNLVPRTES-NH_2
GF17 (Reference)	GFKRIVQRIKDFLRNLV-NH_2
GF19	GFKRIVQRIKDFLRNLVKL-NH2
GF19-FITC	FITC-Ahx-GFKRIVQRIKDFLRNLVKL-NH2

Supplementary Table 5. Theoretical molecular ions (m/z) for mass spectrometry characterization of the synthesized peptides. Mass spectra are shown in Supplementary Fig. 8 below.

	GF19	GF19-FITC
**$[M+2H]^{2+}$**	1172.3	1423.8
**$[M+3H]^{3+}$**	781.8	949.5
**$[M+4H]^{4+}$**	586.6	712.4

Supplementary Table 6. Comparisson of size, zeta potential, and encapasulation efficiency of SiNP and SiNP-GF19.

**Sample**	**SiNP**	**SiNP-GF19**
Size (diam in nm)	131.5 ± 68.8	165.3 ± 64.5
Zeta potential	-21.57 ± 0.84	-15.05 ± 1.11
Encapsulation efficiency (EE)	n/a	96.60 ± 1.17 %.

Supplementary Figures

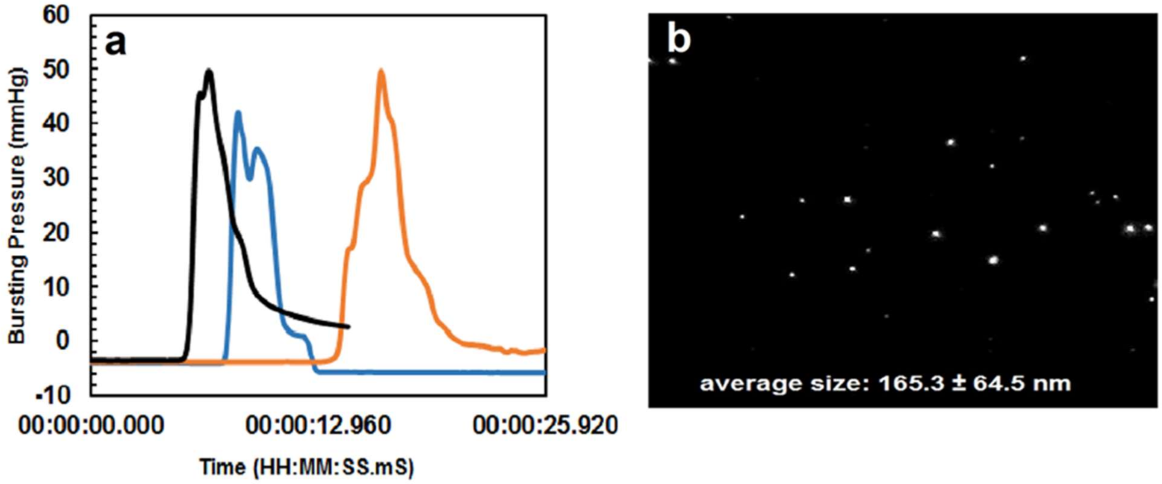

Supplementary Fig. 1. Properties of CIS hydrogels and GF19-containing nanoparticles. a Bursting pressure measurements of three separate CLP-LCPP/collagen-citrate sealant filled excised pig corneas. **b** Zeta-View image of SiNP-GF19.

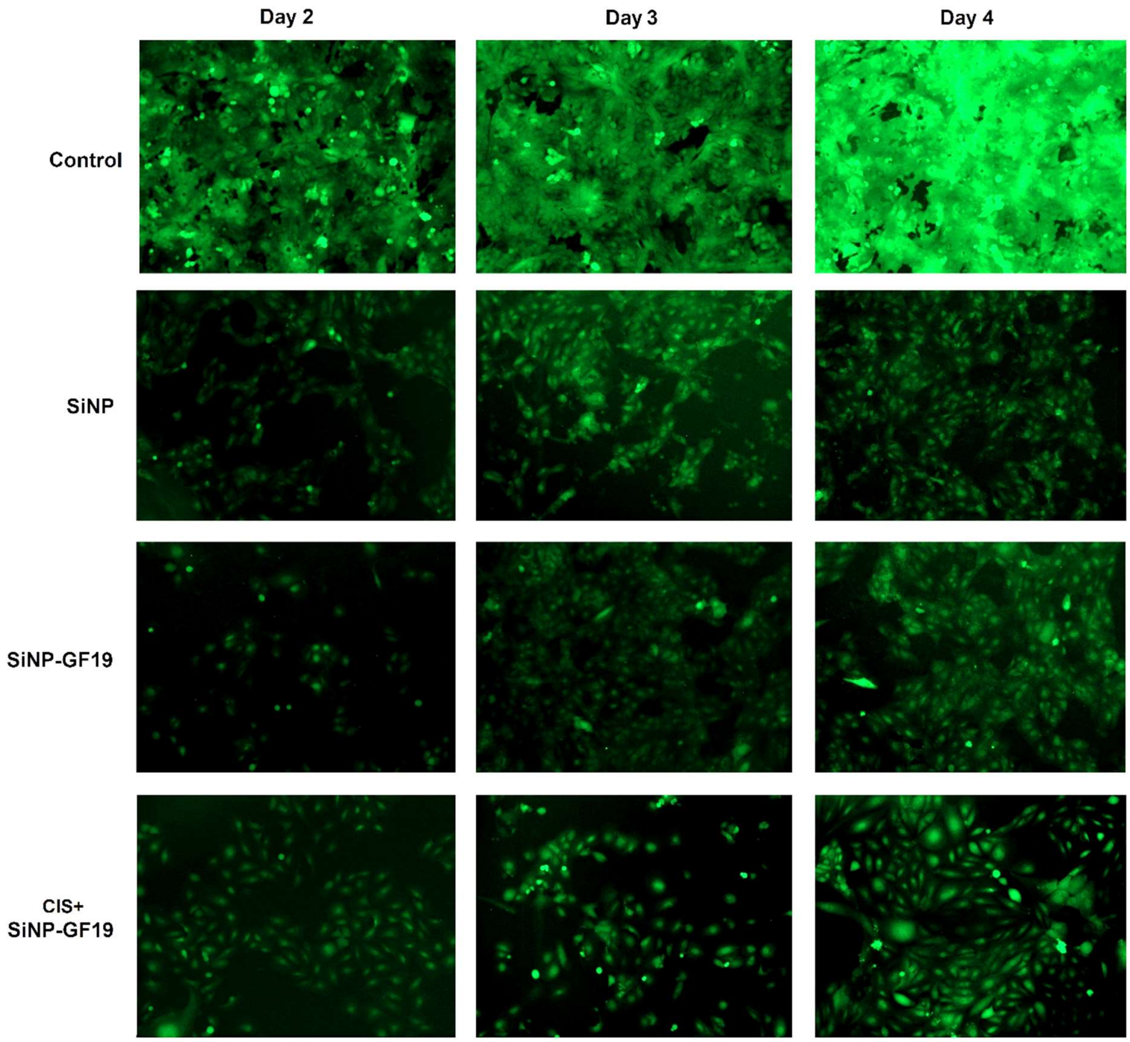

Supplementary Fig. 2. In vitro biocompatibility of CIS and SiNP-GF19. Human corneal epithelial cells cultured in the presence of SiNP, SiNP-GF19 and CIS hydrogels containing SiNP-GF19 after 48, 72, and 96 hours in culture. Controls comprised untreated cells growing on tissue culture plastic.

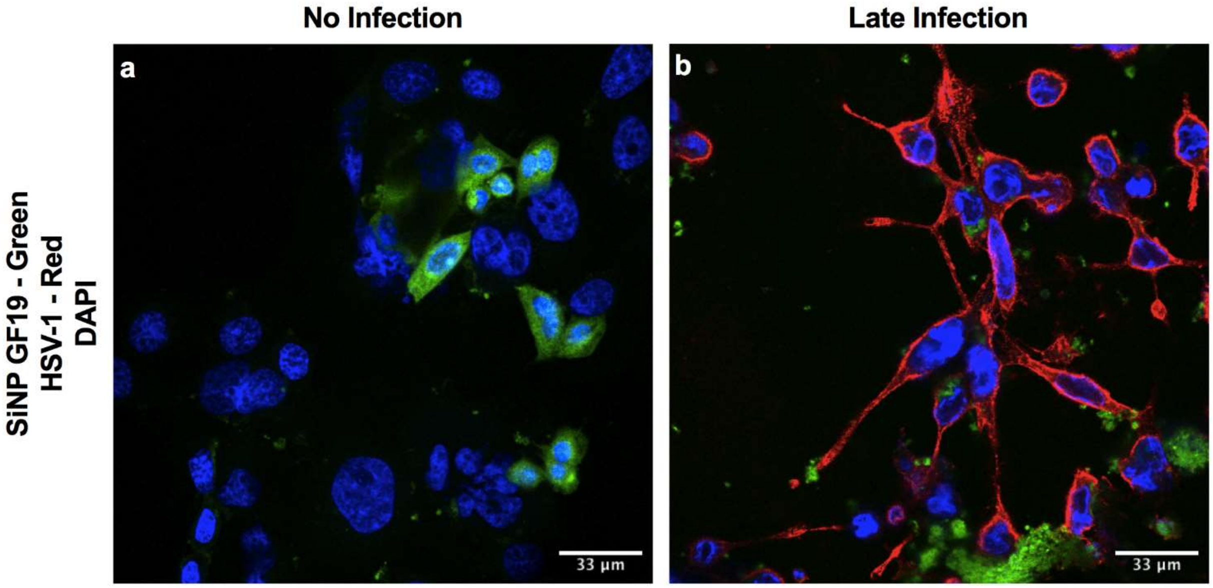

Supplementary Fig. 3. Controls for in vitro testing of GF19. a Uninfected human corneal epithelial cells (HCECs) incubated with SiNP-GF19, showing uptake of green-fluorescently labelled FITC-GF19 into several cells that remained attached and spread. **b** Cultured HCECs simultaneously inoculated with HSV-1 (red fluorescence) and treated with SiNP-GF19, showing both rounded and attached cells. Scale bars, 33 µm.

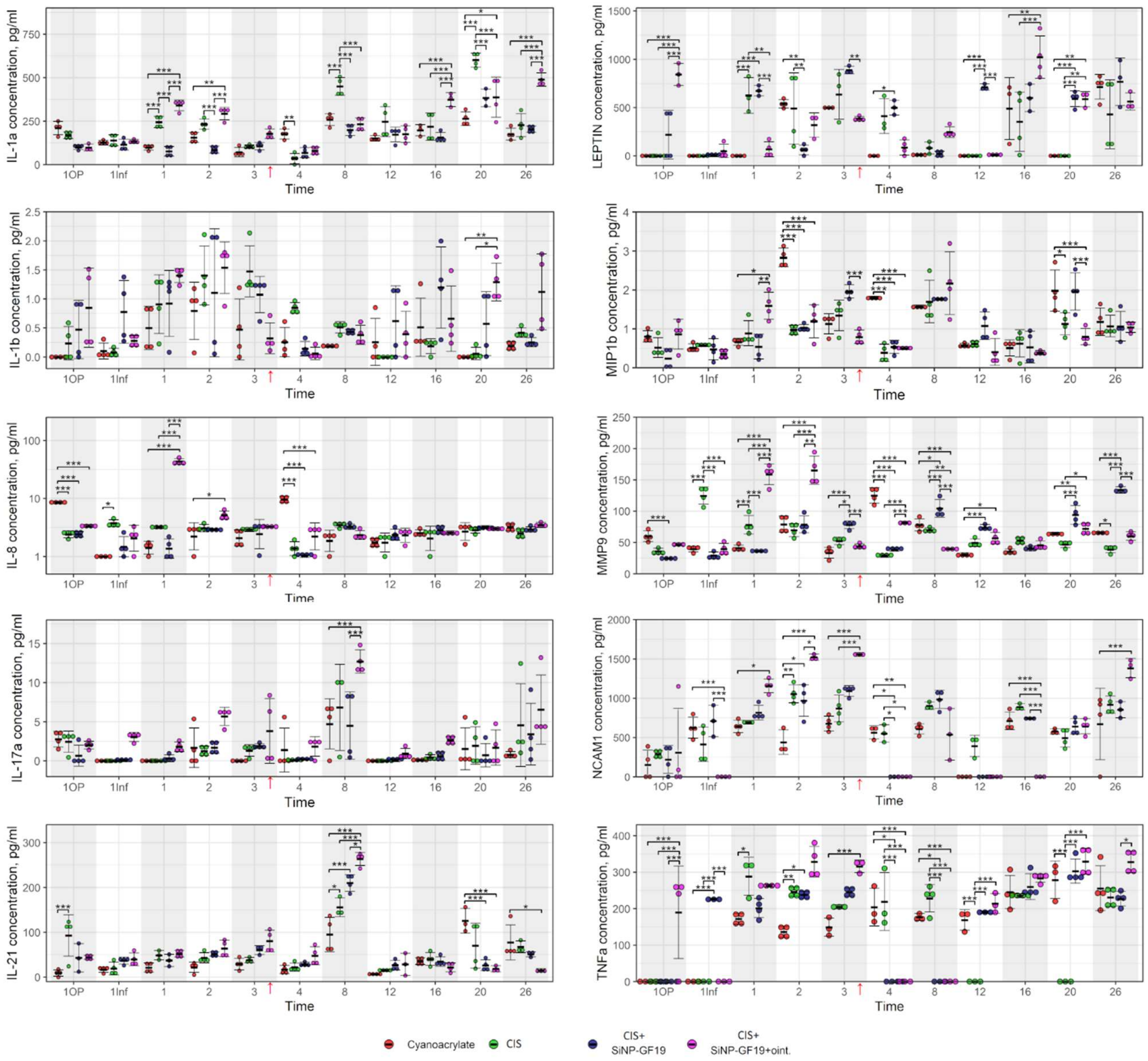

Supplementary Fig. 4. Temporal variation in the concentration of inflammatory cytokines in the tears of eyes with surgically perforated, HSV-1 infected corneas. Inflammatory cytokine levels were evaluated at different time points: 1OP - day after cornea surgery on the left eyes, 1Inf - 1 day after inoculation with 10^4 PFU HSV-1, and 1, 2, 3, 4, 8, 12, 16, 20, and 26 weeks after left eye surgery. Data are represented by dot plots, where the horizontal dash denotes the group mean, and the error bars denote the standard deviation. *, ** and *** denote statistically significant differences between groups at $P \leq 0.05$, $P \leq 0.01$ and $P \leq 0.001$, respectively. The red arrow in the graphs indicates the time point at which the left eyes of the animals in the CIS+SiNP-GF19+oint. group were given GF19 ointment. n=4 technical repeats of 3 pooled samples from each group. The data was assessed using two-way ANOVA with the Tukey HSD post hoc test for multiple comparisons. Statistical reports are provided in Supplementary Table 2.

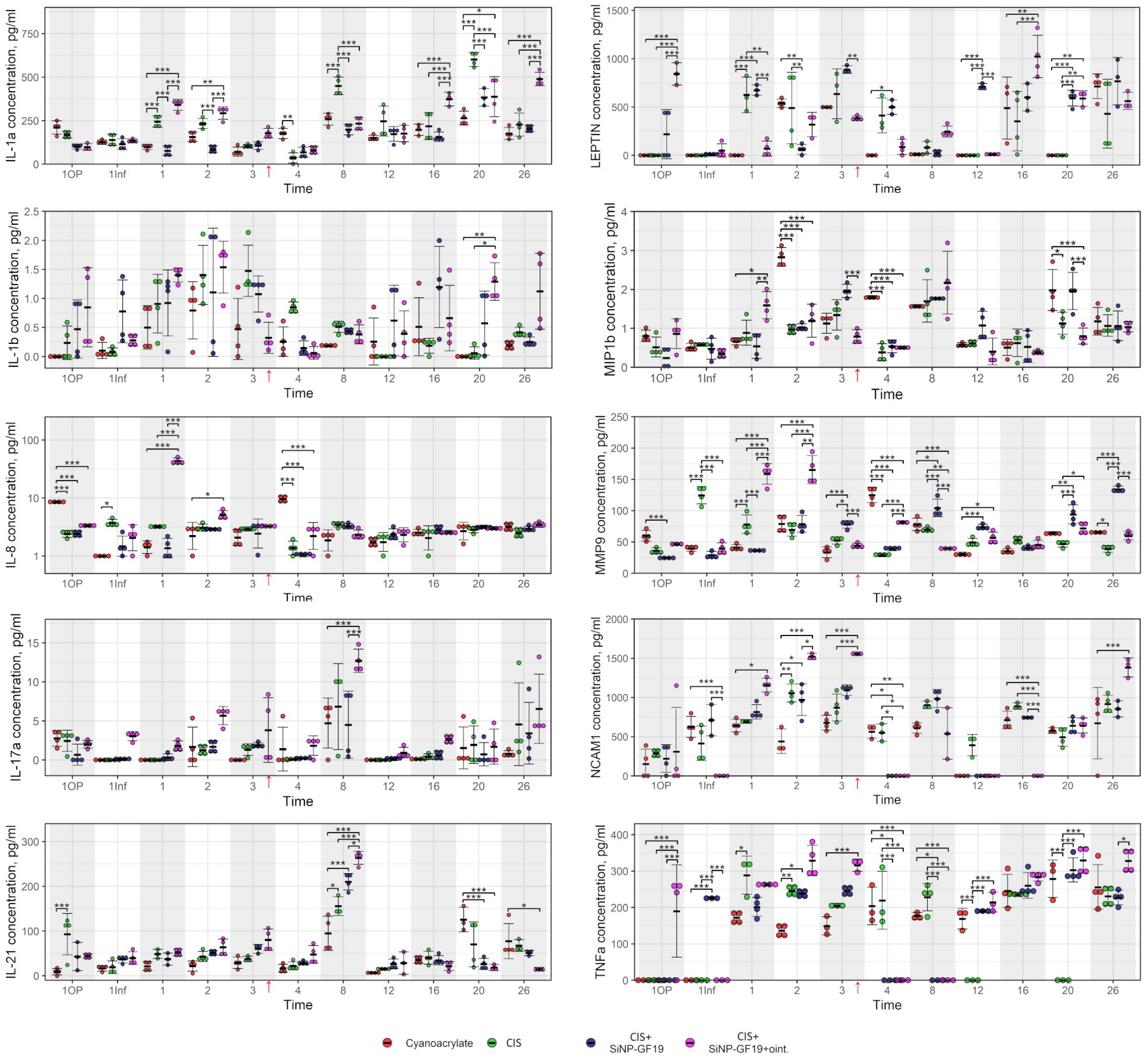

Supplementary Fig. 5. Temporal variation in the concentration of inflammatory cytokines in the tears of the untreated contralateral eyes of rabbits. Inflammatory cytokine levels from tear collected at different time points from the right, untreated eyes: 1OP - day after left eye surgery, 1Inf - 1 day after left eye infection, 1, 2, 3, 4, 8, 12, 16, 20 and 26 weeks after left eye surgery. Data are represented by dot plots, where the horizontal dash denotes the group mean, the error bars denote the standard deviation, and the dots denote the technical repetitions. *, ** and *** * denote statistically significant difference between groups at P ≤ 0.05, P ≤ 0.01 and P ≤ 0.001 respectively. The red arrow in the graphs indicates the time point at which the left eyes of the animals in the CIS+SiNP-GF19+oint group were started to receive the antiviral ointment (oint.) n=4 technical repeats of 3 pooled samples from each group. The data was assessed using two-way ANOVA with the Tukey HSD post hoc test for multiple comparisons. Statistical reports are provided in Supplementary Table 3.

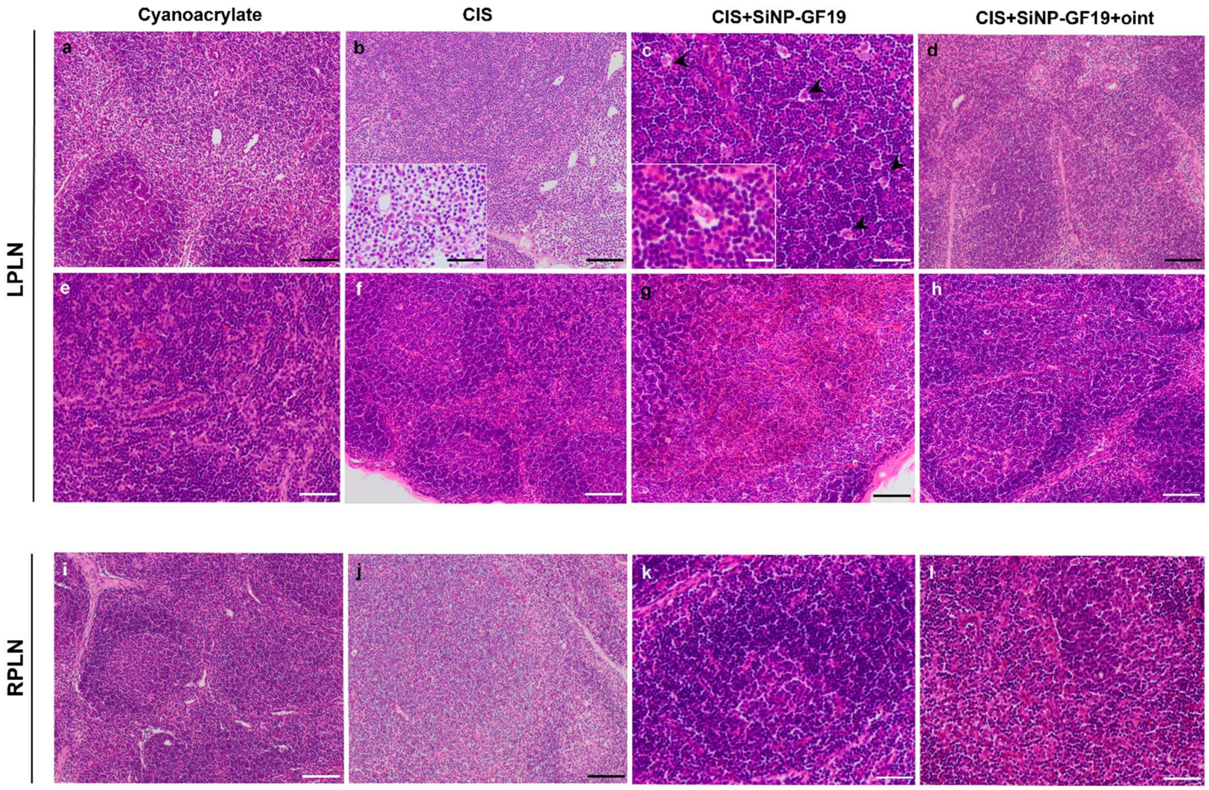

Supplementary Fig. 6. Representative sections of the left and right draining parotid lymph nodes of treated (left) and untreated (right) corneas. a Left parotid lymph node (LPLN) from the cyanoacrylate group showing mild lymphoid depletion. **b** LPLN from LiQD Cornea group with moderate lymphoid depletion, as seen in the inset. **c** LPLN from LiQD Cornea SiNP-GF19 group with low to moderate numbers of tingible body macrophages (arrowheads and inset). **d** LPLN from LiQD Cornea SiNP-GF19 + oint. group with a moderate degree of depletion. **e,f** LPLN from cyanoacrylate and LiQD cornea groups respectively, without histopathological changes. **g** LPLN from LiQD Cornea + SiNP-GF19 showing hemorrhage in the parenchyma. **h** LPLN from LiQD Cornea SiNP-GF19 + oint. without histopathological changes. **i** Right parotid lymph node (RPLN) from cyanoacrylate group showing mild lymphoid depletion. **j** RPLN with a moderate degree of lymphoid depletion. **k,l** RPLN in both groups with SiNP-GF19 without histopathological changes. Scale bars are indicated on each panel. Scale bar in (**a, b, d, f-j**) represents 100 µm; (**c, e, k, l**) - 50 µm. Scale bar in inset from (**b**) represents 50 µm and (**c**) 20 µm.

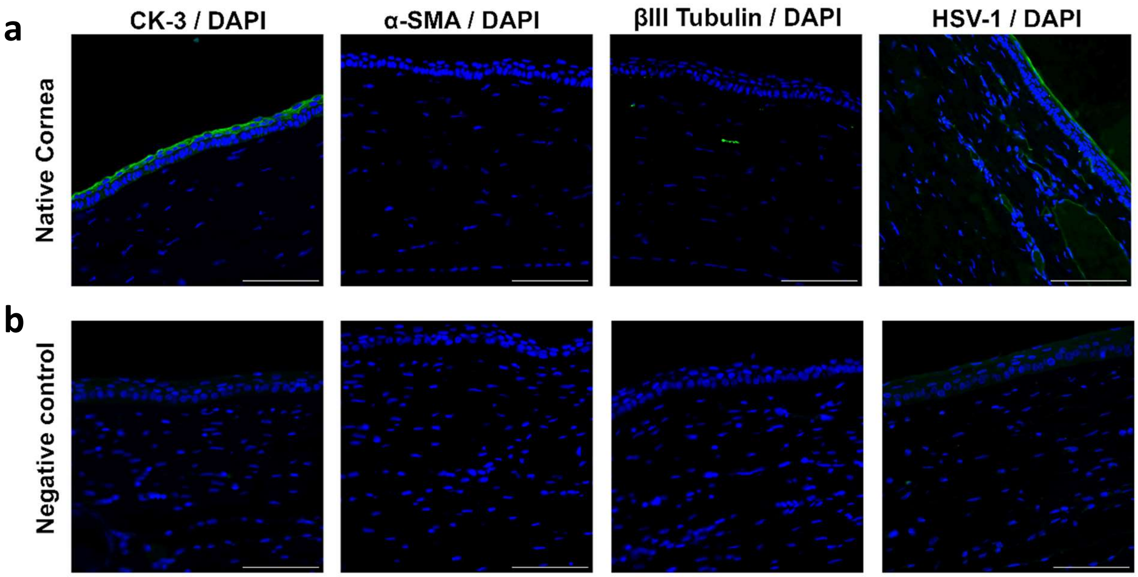

Supplementary Fig. 7. Immunohistochemistry controls. a Control healthy rabbit corneas stained with different antibodies, and **b** negative staining controls where primary antibodies were omitted. Scale bars, 100 μm.

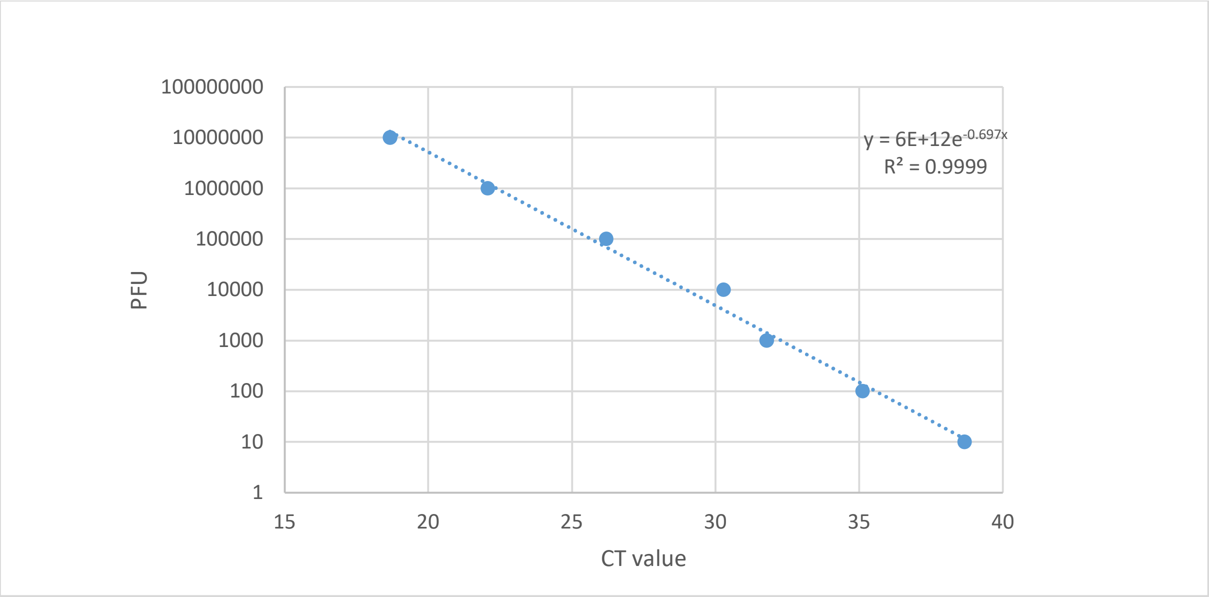

Supplementary Fig. 8. Calibration curve for HSV-1 concentrations for qPCR. qPCR reactions were carried out with known concentrations of HSV-1 virus. Using determined CT values, the PFU of HSV-1 in each sample was obtained.

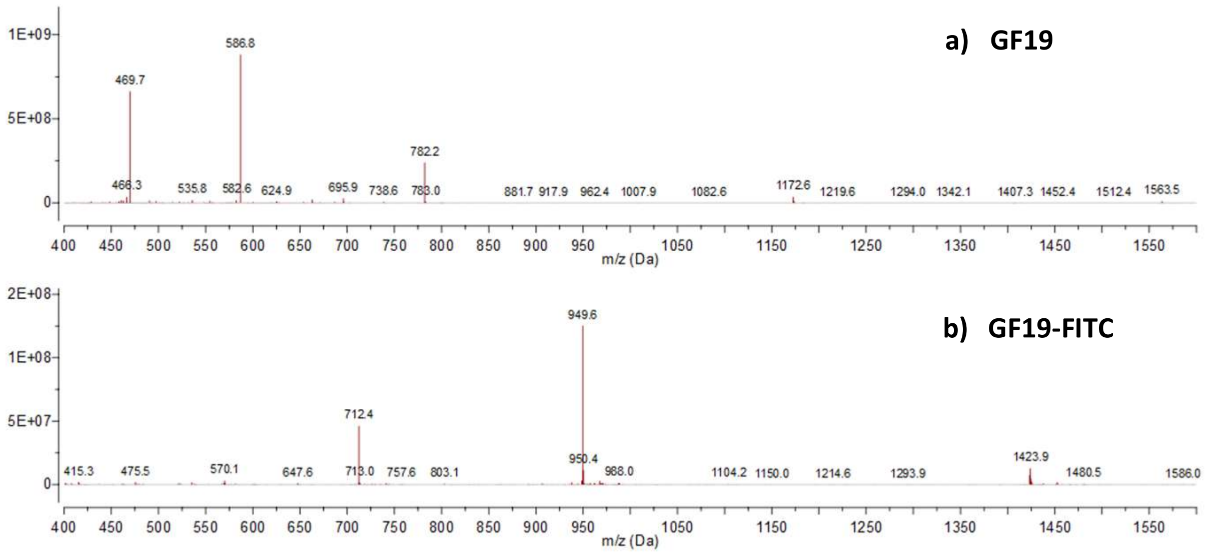

Supplementary Fig. 9. Mass spectra in ESI positive mode of the purified peptides. Purified peptides are analyzed by UPLC-MS in positive ESI mode on independent runs after purification. The confirmation of each ESI ionized peptide mass (**a** for GF19 and **b** for GF19-FITC) matches the theoretical values described in Supplementary Table 5.
