# Supplementary material for: Inflammation-suppressing cornea-in-a-syringe with anti-viral GF19 peptide promotes regeneration in HSV-1 infected rabbit corneas
Source: NPJ Regen Med. 2024 Mar 1;9:11. doi: 10.1038/s41536-024-00355-1 (PMC10907611; doi:10.1038/s41536-024-00355-1)
Supplement: Supplementary file 2 — Reporting summary [file 41536_2024_355_MOESM2_ESM.pdf]

nature portfolio

Corresponding author(s): May Griffith, Virginija Bukelskiene, Jaime Gómez-Laguna, Miguel González-Andrades

Last updated by author(s): Dec 12, 2023

Reporting Summary

Nature Portfolio wishes to improve the reproducibility of the work that we publish. This form provides structure for consistency and transparency in reporting. For further information on Nature Portfolio policies, see our Editorial Policies and the Editorial Policy Checklist.

Statistics

For all statistical analyses, confirm that the following items are present in the figure legend, table legend, main text, or Methods section.

n/a	Confirmed	
☐	☒	The exact sample size (n) for each experimental group/condition, given as a discrete number and unit of measurement
☐	☒	A statement on whether measurements were taken from distinct samples or whether the same sample was measured repeatedly
☐	☒	The statistical test(s) used AND whether they are one- or two-sided<br>*Only common tests should be described solely by name; describe more complex techniques in the Methods section.*
☒	☐	A description of all covariates tested
☐	☒	A description of any assumptions or corrections, such as tests of normality and adjustment for multiple comparisons
☐	☒	A full description of the statistical parameters including central tendency (e.g. means) or other basic estimates (e.g. regression coefficient) AND variation (e.g. standard deviation) or associated estimates of uncertainty (e.g. confidence intervals)
☐	☒	For null hypothesis testing, the test statistic (e.g. F, t, r) with confidence intervals, effect sizes, degrees of freedom and P value noted<br>*Give P values as exact values whenever suitable.*
☒	☐	For Bayesian analysis, information on the choice of priors and Markov chain Monte Carlo settings
☒	☐	For hierarchical and complex designs, identification of the appropriate level for tests and full reporting of outcomes
☒	☐	Estimates of effect sizes (e.g. Cohen's d, Pearson's r), indicating how they were calculated

Our web collection on statistics for biologists contains articles on many of the points above.

Software and code

Policy information about availability of computer code

Data collection	Zen Blue, Carl Zeiss Microscopy, Göttingen, Germany<br>Zeta view View Z-NTA nanoparticle tracking analyzer PMX 120-12C-R4, Particle Metrix, Ammersee, Germany<br>RP-UPLC-UV/MS in a Waters Acquity UPLC Xevo TQD,Waters Corp. Milford, MA, USA<br>Differential Scanning Calorimeter DSC 25, TA Instruments – Waters LLC, New Castle, DE, USA<br>Fourier-transform Infrared spectroscopy (FTIR-ATR), Thermo Scientific, Nicolet 6700 / Smart iTR, Waltham, MA, USA<br>Bruker Avance III HD 600 MHz NMR Spectrometer, Bruker, Billerica, MA, USA<br>Bruker D8 Advance XRD, Bruker, Billerica, MA, USA<br>QuantStudio™ 3 Real-Time PCR system, Thermo Fisher Scientific, Waltham, MA, USA<br>InnoScan 710 microarray scannng system, Innopsys, Carbonne, France
Data analysis	R statistical software, version 4.1.2 was used for statistical analysis of all data and plotting of graphs;<br>FIJI, Schindelin, J.; Arganda-Carreras, I. & Frise, E. et al. (2012), "Fiji: an open-source platform for biological-image analysis", Nature methods 9(7): 676-682<br>Zeta View Nanoparticle Tracker software - ZetaView version 8.05.16 SP3.

For manuscripts utilizing custom algorithms or software that are central to the research but not yet described in published literature, software must be made available to editors and reviewers. We strongly encourage code deposition in a community repository (e.g. GitHub). See the Nature Portfolio guidelines for submitting code & software for further information.

Data

Policy information about availability of data

All manuscripts must include a data availability statement. This statement should provide the following information, where applicable:

- Accession codes, unique identifiers, or web links for publicly available datasets
- A description of any restrictions on data availability
- For clinical datasets or third party data, please ensure that the statement adheres to our policy

Provide your data availability statement here.

Research involving human participants, their data, or biological material

Policy information about studies with human participants or human data. See also policy information about sex, gender (identity/presentation), and sexual orientation and race, ethnicity and racism.

Reporting on sex and gender	N/A
Reporting on race, ethnicity, or other socially relevant groupings	N/A
Population characteristics	N/A
Recruitment	N/A
Ethics oversight	N/A

Note that full information on the approval of the study protocol must also be provided in the manuscript.

Field-specific reporting

Please select the one below that is the best fit for your research. If you are not sure, read the appropriate sections before making your selection.

☒ Life sciences ☐ Behavioural & social sciences ☐ Ecological, evolutionary & environmental sciences

For a reference copy of the document with all sections, see nature.com/documents/nr-reporting-summary-flat.pdf

Life sciences study design

All studies must disclose on these points even when the disclosure is negative.

Sample size	This was an exploratory study using the Resource Equation method for sample size determination (Festing MFW, Altman DG, D. G. (2002). Guidelines for the Design and Statistical Analysis of Experiments Using Laboratory Animals. ILAR Journal 2002; 43(4), 244–258; Arifin WN, Zahiruddin WM. Sample Size Calculation in Animal Studies Using Resource Equation Approach. Malays J Med Sci. 2017;24(5):101-105). Using a group comparison design with repeated measures, with one within and one between group factors, the sample size needed is 6 animals per group.
Data exclusions	Three animals died prematurely. However, tissue was still collected and analysed by necropsy.
Replication	Each animal was considered an experimental unit, N=6 rabbits per group. Each animal received a separately prepared test article.
Randomization	Allocation of animals to groups was random and performed by animal facility staff upon receipt.
Blinding	Complete blinding was not possible because the different groups received different treatments that were very obvious in the transparent cornea (e.g., one group had extra ointment, and cyanoarylate glue-treated corneas are very obvious). However, 2-3 different personnel collecting the data and performing the analyses were not told the contents of the samples nor what to expect. The histopathology and immunohistochemistry were done by a different group (in a different country) in this transnational study and the two vet pathologists were blinded.

Reporting for specific materials, systems and methods

We require information from authors about some types of materials, experimental systems and methods used in many studies. Here, indicate whether each material, system or method listed is relevant to your study. If you are not sure if a list item applies to your research, read the appropriate section before selecting a response.

Materials & experimental systems

n/a	Involved in the study
☐	☒ Antibodies
☐	☒ Eukaryotic cell lines
☒	☐ Palaeontology and archaeology
☐	☒ Animals and other organisms
☒	☐ Clinical data
☒	☐ Dual use research of concern
☒	☐ Plants

Methods

n/a	Involved in the study
☒	☐ ChIP-seq
☒	☐ Flow cytometry
☒	☐ MRI-based neuroimaging

Antibodies

Antibodies used

Mouse anti-CK-3 monoclonal antibody (Abcam ab68260)
Mouse anti-α-SMA monoclonal antibody (Invitrogen MA5-11547)
Mouse anti-βIII tubulin monoclonal antibody (Invitrogen MA1-118)
Goat anti-HSV type 1 polyclonal antibody (Invitrogen PA1-7493)
Anti-mouse Alexa488-conjugated secondary antibody (Invitrogen A-11001)
Anti-goat Alexa488-conjugated secondary antibody (Invitrogen A-11055)
Donkey anti-goat, DyLight™ 550, Invitrogen SA510087

Validation

Validation information from the manufacturer:
Mouse anti-CK-3 (concentration of 0.25 μg/ml)
mouse anti-α-SMA (1:100-1:500)
Mouse anti-βIII tubulin (1:50-1:200)
Goat anti-HSV type 1 (1:4000)
Anti-mouse Alexa488 (A-11001: 1ug/ml)
Anti-goat Alexa488 (A-11055: 1-10ug/ml)
anti-goat, DyLight™ 550 (1:50-1:500)

Eukaryotic cell lines

Policy information about cell lines and Sex and Gender in Research

Cell line source(s)

SV40-immortalized human corneal epithelial cells (HCECs) were a gift from H Handa, Division of Ophthalmology, Kinki Central Hospital, Hyogo, Japan. These cells retained many key characteristics of primary human corneal epithelial cells.

Authentication

The initial immortalized HCEC line was characterized using the expression of keratin and large T antigen (Araki-Sasaki et al. Invest Ophthalmol. Vis. Sci. 1995;36(3):614-621.) GFP-HCECs were subsequently characterized by morphology, and expression of Integrin Beta1 and focal adhesion kinase cell proliferation rate (Islam et al. Acta Biomater. 2015; 12:70-80.). We previously showed that HCECs were susceptible to HSV-1 infection and could recover (Lee et al. Trans. Vis. Sci. Tech. 2014; 3(3):4. https://doi.org/10.1167/tvst.3.3.4.).

Mycoplasma contamination

These cells were not tested for mycoplasma contamination.

Commonly misidentified lines
(See ICLAC register)

N/A

Animals and other research organisms

Policy information about studies involving animals; ARRIVE guidelines recommended for reporting animal research, and Sex and Gender in Research

Laboratory animals

New Zealand White rabbits, male animals - 3.2 to 4.2 kg in weight.

Wild animals

N/A

Reporting on sex

As described in the MS, HSV-1 infects both males and females equally (Riccio, R.E., Park, S.J., Longnecker, R. & Kopp, S.J. Characterization of sex differences in ocular herpes simplex virus 1 infection and herpes stromal keratitis pathogenesis of wild-type and herpesvirus entry mediator knockout mice. Msphere 4, 10.1128/msphere. 00073-00019 (2019)). For the study 24 male New Zealand white rabbits were used as they were more docile for eye examinations.

Field-collected samples

N/A

Ethics oversight

We have complied with all relevant ethical regulations. For the animal (rabbit) study, ethical permission was obtained from the The study was performed after approval from the Lithuanian State Food and Veterinary Service, permit no. G2-179; 2021-05-28. The guidelines of the Association for Research in Vision and Ophthalmology (ARVO) were followed.

Note that full information on the approval of the study protocol must also be provided in the manuscript.

Plants

Seed stocks	N/A
Novel plant genotypes	N/A
Authentication	N/A
